# Integrated microRNA and mRNA signatures associated with overall survival in epithelial ovarian cancer

**DOI:** 10.1371/journal.pone.0255142

**Published:** 2021-07-28

**Authors:** Joanna Lopacinska-Jørgensen, Douglas V. N. P. Oliveira, Guy Wayne Novotny, Claus K. Høgdall, Estrid V. Høgdall

**Affiliations:** 1 Department of Pathology, Herlev University Hospital, Herlev, Denmark; 2 Department of Gynaecology, Juliane Marie Centre, Rigshospitalet, University of Copenhagen, Copenhagen, Denmark; Universitat des Saarlandes, GERMANY

## Abstract

Ovarian cancer (OC), the eighth-leading cause of cancer-related death among females worldwide, is mainly represented by epithelial OC (EOC) that can be further subdivided into four subtypes: serous (75%), endometrioid (10%), clear cell (10%), and mucinous (3%). Major reasons for high mortality are the poor biological understanding of the OC mechanisms and a lack of reliable markers defining each EOC subtype. MicroRNAs (miRNAs) are small non-coding RNA molecules that regulate gene expression primarily by targeting messenger RNA (mRNA) transcripts. Their aberrant expression patterns have been associated with cancer development, including OC. However, the role of miRNAs in tumorigenesis is still to be determined, mainly due to the lack of consensus regarding optimal methodologies for identification and validation of miRNAs and their targets. Several tools for computational target prediction exist, but false interpretations remain a problem. The experimental validation of every potential miRNA-mRNA pair is not feasible, as it is laborious and expensive. In this study, we analyzed the correlation between global miRNA and mRNA expression patterns derived from microarray profiling of 197 EOC patients to identify the signatures of miRNA-mRNA interactions associated with overall survival (OS). The aim was to investigate whether these miRNA-mRNA signatures might have a prognostic value for OS in different subtypes of EOC. The content of our cohort (162 serous carcinomas, 15 endometrioid carcinomas, 11 mucinous carcinomas, and 9 clear cell carcinomas) reflects a real-world scenario of EOC. Several interaction pairs between 6 miRNAs (hsa-miR-126-3p, hsa-miR-223-3p, hsa-miR-23a-5p, hsa-miR-27a-5p, hsa-miR-486-5p, and hsa-miR-506-3p) and 8 mRNAs (*ATF3*, *CH25H*, *EMP1*, *HBB*, *HBEGF*, *NAMPT*, *POSTN*, and *PROCR*) were identified and the findings appear to be well supported by the literature. This indicates that our study has a potential to reveal miRNA-mRNA signatures relevant for EOC. Thus, the evaluation on independent cohorts will further evaluate the performance of such findings.

## Introduction

Ovarian cancer (OC) is the eighth leading cause of cancer-related death among females worldwide [1]. The high mortality of epithelial OC (EOC) patients is related to asymptomatic and hidden growth of the tumor thus reflecting on the fact that the detection is often in late stages of the diseases [2]. Approximately two‐thirds of all EOC patients are diagnosed in late FIGO stages: III and IV according to the International Federation of Gynecology and Obstetrics [3]. The 5-year survival rate of stage IV patients is less than 30%, whereas that of patients in stage I is higher than 90% based on the data from Danish Gynecologic Cancer Database (DGCD) [4]. The heterogeneity of OC accounts for the high mortality rates [5]. Epithelial OC (EOC), which comprises 90–95% of the OC cases [6, 7], can be divided into four major types: serous (75%), endometrioid (10%), clear cell (10%), and mucinous (3%) [8]. Despite massive effort, no effective screening method for EOC yet exists [9].

MicroRNAs (miRNAs) are small non-coding RNA molecules involved in the transcriptional and post-transcriptional regulation of gene expression. Although numerous studies have indicated their potential as disease biomarkers in different human cancer, including OC [6, 10–13], these molecules are still not used in a routine testing. The results generated by various miRNA studies are not invariably consistent, as there is a lack of consensus regarding optimal methodologies for performing miRNA detection, data analysis and standardization [14–18]. In regard to OC, the limited overlap is also due to the differences in specimen heterogeneity and study design, as some studies compared miRNA expression in ovarian serous adenocarcinoma tissues to normal ovary [19], whereas others investigated the differences between recurrent versus primary OC tissue [20]. Moreover, to understand the role of a miRNA, its target genes and binding sites need to be determined. The identification of miRNA targets remains challenging, as miRNA-mRNA base pairing is not perfectly complementary and one miRNA can target multiple genes, whereas one gene can be targeted by many miRNAs [15–17].

MiRNAs have long been recognized for negative regulation of target mRNA, however there is growing evidence that miRNA can also promote gene expression *via* different mechanisms, including miRNA-host gene co-expression, inhibiting upstream suppressor, co-regulation by shared transcription factors, and targeting gene enhancers [21, 22]. For instance, by binding to gene promoters, hsa-mir-373 was reported to activate E-cadherin and cold-shock domain-containing protein C2 [23] and hsa-miR-205 was shown to induce the expression of the interleukin (IL) tumor suppressor genes *IL24* and *IL32* [24]. *FBP1* and *FANCC* were activated by hsa-miR-24-1 targeting gene enhancers [25].

To exploit miRNAs as biomarkers in EOC, deeper understanding of their expression and potential interaction with mRNAs is necessary. Therefore, the aim of this study was to perform an integrative correlation analysis of miRNA and mRNA expression profiles data associated with overall survival (OS) of EOC patients. OS is considered as the gold standard in oncology research, especially in more aggressive forms of disease with short life expectancy. Furthermore, given that both mRNA and miRNA have been shown to be associated with OS, often in individual studies and methodologies, our current study aimed at clarifying their possible correlation in association with OS. We employed the same platform and data treatment workflow (i.e. microarray-based, data background correction, and normalization), in order to strengthen the evaluation by minimizing methodological variances. Moreover, we further investigated whether miRNA-mRNA signatures might be useful to distinguish between different subtypes of EOC.

## Materials and methods

### Patients and samples collection

All tissue samples were obtained from patients enrolled in the Danish Pelvic Mass study, as described previously [26–28]. Patients were diagnosed and surgically treated for EOC between October 2004 and January 2010. All patients were registered in the Danish Gynecologic Cancer Database (DGCD) [29], a national mandatory clinical database, as well as in the Bio- and Genome Bank, Denmark (RBGB,www.regioner.dk), a registry mainly including clinical biobanks, ensuring biological material of high quality for patients own treatment and biomarker research.

Tumor tissues stored as formalin-fixed and paraffin embedded (FFPE) were used in this study. All histologic diagnoses were performed by a pathologist specialized in gynecology. Primarily, 246 patients with EOC were identified and considered for inclusion in the study. From those, 49 subjects were excluded due to either insufficient tumor material for analysis (n = 24), neoadjuvant chemotherapy or ongoing palliative care (n = 15), other forms of cancer (n = 8), or ambiguous histologic classification (n = 2). Finally, 197 patients (162 serous carcinomas, 15 endometrioid carcinomas, 11 mucinous carcinomas, and 9 clear cell carcinomas) were eligible for data analysis. All samples in this study showed a tumor presence above 50% based on conventional hematoxylin and eosin staining.

The study was performed according to the guidelines of the Declaration of Helsinki, including written informed consent from all patients, and it has been approved by the Danish National Committee for Research Ethics, Capital Region (approval codes KF01-227/03 and KF01-143/04). Patients were followed from October 2004 until January 17th, 2015, five years after the last patient was included, and none were lost to follow-up. Median follow-up was 88 months, with the shortest follow-up time for a patient still alive on 61 months. At the end of follow-up, 133 (67.5%) patients had died, and 64 (32.5%) patients were still alive.

### MicroRNA and mRNA microarray profiling

Total RNA was extracted from 20μm thick FFPE tumor sections using the RecoverAll Total Nuclei Acid Isolation Kit for FFPE samples (Ambion, USA). Samples were then hybridized to either Affymetrix GeneChip miRNA 2.0 Array (Affymetrix, USA) for miRNA profiling or to Affymetrix GeneChip Human Genome U133 Plus 2.0 Array (Affymetrix, USA) for gene profiling. Hybridization was performed according to the manufacturer instructions. Microarrays were scanned in a Genechip Scanner (Affymetrix, USA), and data acquisition was performed by GeneChip Command Console (Affymetrix, USA).

### Data treatment and statistical analysis

The miRNA and mRNA raw data were separately processed by background-correction, normalization, and log-transformation by applying the robust multi-array average (RMA) method [30], resulting in 854 miRNA and 54,612 mRNA probes, respectively. MiRNA and mRNA candidate targets were identified in separate univariate and multivariable Cox regression analyses of OS, defined as time in months, counting from the time of diagnosis (surgery) to time of death, or last censored follow-up. In the first step, each of the miRNAs or mRNAs was submitted to univariate Cox regression analysis. Due to the large number of predictors, a lasso (least absolute shrinkage and selection operator) penalized model for Cox multivariate regression was applied and the resulting targets were finally cross-validated (10-fold) by a last round of Cox multivariate analysis. Pearson correlation test was used to explore pair-wise correlations between identified miRNAs and mRNAs targets. All miRNA-mRNA pairs with P< 0.05 and coefficient (R) below -0.4 or above 0.4 were considered for further analysis. All statistical analyses were performed in the R environment [31].

### Construction of the miRNA-target regulatory network

All candidate correlations found were further submitted to functional validation analysis using miRTarBase v8.0 database [32, 33] and the R package multiMiR [34], comprising a compilation of 14 miRNA-mRNA data resources, such TarBase 8.0 [35], miRDB v6 [36], TargetScan v7.2 [37], DIANA-microT-CDS v5 [38], and others. All miRNAs were annotated according to the current version of miRbase (version 22) [39]. The relevant literature given as an output from databases search was manually surveyed to ensure collection of strong evidence of experimentally validated miRNA-mRNA interactions.

To gain insight into the functions of selected miRNA-mRNA interactions, we performed Gene Ontology (GO) classification and pathway analysis by Kyoto Encyclopedia of Genes and Genomes (KEGG) with DIANA-miRPath v3.0 online tool (www.microrna.gr) [40]. The network of miRNA-mRNA interactions was visualized by Cytoscape [41].

## Results

### Patients

Tissues from a total of 197 patients with EOC were included in this study with following histological subtypes: 162 (82.2%) serous adenocarcinomas, 15 (7.6%) endometrioid adenocarcinomas, 11 (5.6%) mucinous adenocarcinomas, and 9 (4.6%) clear cell carcinomas. 52 (26.4%) of the cases were early-stage diagnoses (FIGO I-II), while 145 (73.6%) were classified as advanced stages (FIGO III-IV). Low-grade tumors accounted for 20 (10.2%) patients, whereas high-grade tumors were found in 177 (89.8%) of the patients. Thirty-nine (19.8%) women were categorized with type I tumor and 158 (80.2%) with type II tumor. Clinical and pathologic information on the patients is summarized in Table 1. The results of multivariate analysis of OS with following clinical features included in the model: stage (early vs late), type (I vs II), treatment response (>60 months before progression of disease vs <60 months before progression of disease), menopause (pre-menopausal vs post-menopausal) are presented in S1 Table.

**Table 1:** Clinicopathological characteristics of 197 epithelial ovarian cancer patients.

Status	
Alive	64 (32.5%)
Death	133 (67.5%)
**Median age in years (range)**	64 (31–89)
**Median OS**^**1**^ **in months**	48 (95% CI: 40–52)
**Histology**	
Serous adenocarcinoma	162 (82.2%)
Mucinous adenocarcinoma	11 (5.6%)
Endometrioid adenocarcinoma	15 (7.6%)
Clear Cell adenocarcinoma	9 (4.6%)
**FIGO**^**2**^ **stage**	
I	31 (15.7%)
II	21 (10.7%)
III	119 (60.4%)
IV	26 (13.2%)
**Histological grade**	
1	20 (10.2%)
2	102 (51.8%)
3	74 (37.6%)
Unknown	1 (< 1%)
**Type I or II**	
I	39 (19.8%)
II	158 (80.2%)

^1^ OS–overall survival

^2^ FIGO–International Federation of Gynecology and Obstetrics

### MiRNAs and mRNAs associated with overall survival

854 miRNAs and 54,612 mRNAs were analyzed as predictor variables of the patients OS. The analysis workflow employed is presented on S1 Fig. Each of the miRNAs or mRNAs was submitted to univariate Cox regression analysis and in total, 36 miRNAs and 1,728 mRNAs were found as potential predictors (P < 0.01). In the next step, the multivariate Cox regression was used to evaluate the combination of all predictors in association with OS. We further implemented a lasso-penalty variation followed by cross-validation (10 iterations). Here, this approach was implemented to improve our model, by accounting for the large number of candidates primarily found and validate it by random iteration, respectively. In total, 17 miRNA and 30 mRNA targets were identified (S2 Table).

### Integrated miRNA and mRNA signatures associated with OS in EOC

Pearson correlation test was used to explore pair-wise correlations between those 17 miRNAs and 30 mRNAs targets. A correlation was considered significant if the correlation coefficient was < -0.4 or > 0.4, and P < 0.05. We found 1 negatively and 11 positively correlated miRNA-mRNA interaction pairs between 6 miRNAs (hsa-miR-126-3p, hsa-miR-223-3p, hsa-miR-23a-5p, hsa-miR-27a-5p, hsa-miR-486-5p, and hsa-miR-506-3p) and 8 mRNAs (*ATF3*, *CH25H*, *EMP1*, *HBB*, *HBEGF*, *NAMPT*, *POSTN*, and *PROCR*) (Table 2 and S2 Fig). To determine the contribution of each miRNA-mRNA pair to OS, we classified the patients into two groups: “high risk” and “low risk” for short OS. In most pairs, except for hsa-miR-506-93~*POSTN*, considering that they presented a positive correlation, a subject presenting the overexpression of both targets (in regard to the respective target median, and hazard ratio, HR, above 1) were classified as “high risk”, otherwise as “low risk”. In the case of hsa-miR-506-3p~*POSTN* pair, subjects with overexpression of *POSTN* and downregulation of hsa-miR-506-3p were classified as “high risk”. The classification showed that “high risk” patients had a significantly lower OS in comparison to the “low risk” patients (p < 0.0001, except hsa-miR-27a-5p~EMP1, where p = 0.0019) (S3 Fig).

**Table 2:** 12 miRNA-mRNA interaction pairs associated with overall survival in epithelial ovarian cancer cohort.

miRNA				mRNA				Pearson correlation		Association ranks and their p-values from Jacobsen et al.’s study	
Target	Median	HR	Std. Error	Target	Median	HR	Std. Error	Cor.	P.val	Association rank in OC	P-value
hsa-miR-126-3p	8.15	1.43	0.10	*PROCR*	4.38	1.50	0.10	0.473	2.64E-12	115	9.40e-08
hsa-miR-223-3p	1.90	1.32	0.10	*HBEGF*	5.80	1.63	0.10	0.421	8.15E-10	395	2.11e-10
hsa-miR-223-3p				*CH25H*	4.47	1.74	0.11	0.404	4.18E-09	428	7.08e-10
hsa-miR-223-3p				*NAMPT*	7.34	1.84	0.12	0.413	1.76E-09	328	2.67e-11
hsa-miR-23a-5p	3.45	1.46	0.09	*ATF3*	7.36	1.39	0.06	0.413	1.82E-09	1651	3.76e-02
hsa-miR-23a-5p				*HBEGF*	5.80	1.63	0.10	0.434	2.04E-10	1092	1.75e-02
hsa-miR-27a-5p	2.29	1.36	0.08	*EMP1*	7.83	1.61	0.08	0.425	5.26E-10	496	5.16e-04
hsa-miR-27a-5p				*ATF3*	7.36	1.39	0.06	0.432	2.51E-10	8	7.85e-10
hsa-miR-27a-5p				*HBEGF*	5.80	1.63	0.10	0.441	1.03E-10	333	1.81e-04
hsa-miR-486-5p	4.59	1.15	0.05	*ATF3*	7.36	1.39	0.06	0.484	6.49E-13	23	3.08e-19
hsa-miR-486-5p				*HBB*	6.83	1.34	0.06	0.737	8.52E-35	2	7.33e-47
hsa-miR-506-3p	1.54	0.85	0.06	*POSTN*	2.85	1.64	0.10	-0.459	1.36E-11	1	1.28e-34

Std. Error: standard error; HR: hazard ratio; Cor.: correlation; P.val: P-value.

Association rank and P-value are from Jacobsen at el. study [42], which is explored further in the Discussion.

**Fig 1:**
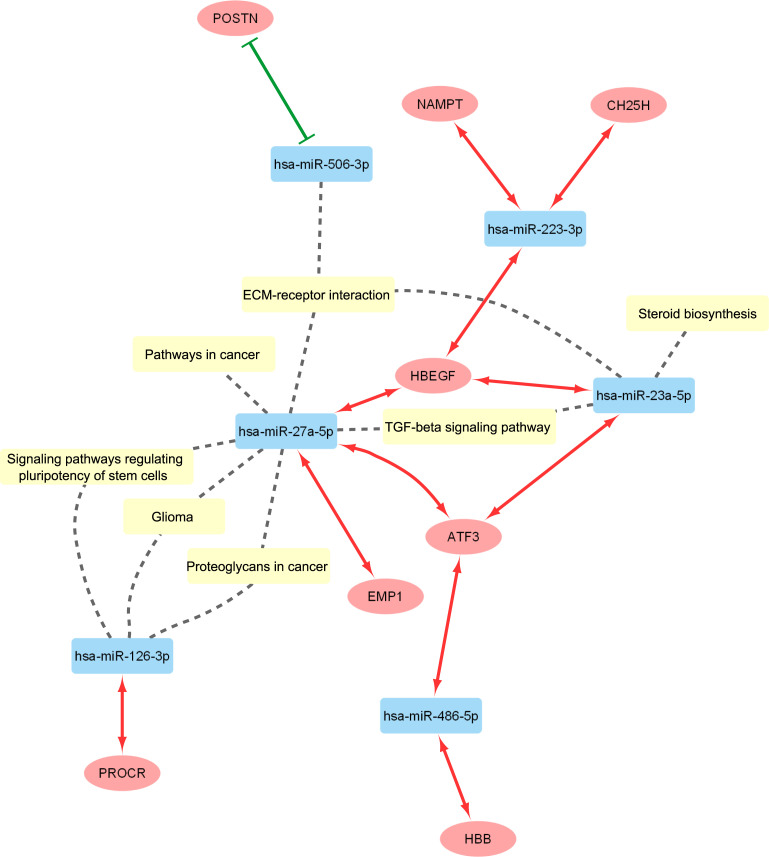
Network analysis of 12 miRNA-mRNA interaction pairs associated with OS in EOC patients. Each blue rectangular node represents a miRNA, whereas each red oval node presents a mRNA. Edge with solid green line indicates negative correlation between a miRNA and a mRNA (P < 0.05, R < -0.4), while red arrow line designates positive correlation (P < 0.05, R > 0.4). Dashed grey lines represent miRNAs being involved in various pathways as indicated by pathway enrichment analysis.

Network and pathway analysis of 12 miRNA-mRNA interactions are presented in Fig 1. Pathway enrichment revealed that 3 miRNAs: hsa-miR-23a-5p, hsa-miR-27a-5p, and hsa-miR-506-3p may contribute to extracellular matrix (ECM)-receptor interaction. Hsa-miR-27a-5p and hsa-miR-126-3p are involved in three processes: signalling pathways regulating pluripotency of stem cells, glioma, and proteoglycans in cancer. Hsa-miR-23a-5p and hsa-miR-27a-5p are linked to transforming growth factor beta (TGF-beta) signaling pathway. Moreover, hsa-miR-23a-5p seems to be important in steroid biosynthesis, whereas hsa-miR-27a-5p is relevant for pathways in cancer.

We further investigated our miRNA-mRNA candidates based on access to different databases, such as miRTarBase v8.0 database [32, 33] and the R package multiMiR [34]. Two out of twelve identified miRNA-mRNA signatures were found by miRNA-target prediction databases: hsa-miR-223-3p~*NAMPT* and hsa-miR-27a-5p~*ATF3*. To our knowledge, no prior experimental studies have directly validated any of our 12 miRNA-mRNA interactions.

### Integrated miRNA-mRNA signatures in EOC subtypes

As 82% of samples in our study (162 out of 197) were derived from serous adenocarcinoma histologic subtype, we sought to investigate whether our results were skewed by this overrepresentation. To that end, those 17 miRNAs and 30 mRNAs targets (S2 Table) originally found in our analysis were further subjected to correlation test but this time in two subgroups: serous adenocarcinoma (n = 162) and non-serous adenocarcinoma (n = 35). We found 19 miRNA-mRNA interaction pairs for serous subtype, and 6 pairs for non-serous subgroup. In the next step, we compared miRNA-mRNA signatures found in each subgroup to 12 miRNA-mRNA interactions identified based on the whole cohort (Table 3). Three pairs, hsa-miR-486-5p~*ATF3*, hsa-miR-486-5p~*HBB*, and hsa-miR-506-3p~*POSTN* were found to be shared between these two subgroups and the total cohort. Interestingly, specific miRNA-mRNA pairs were discovered for both subtypes: six for serous adenocarcinoma (hsa-miR-23a-5p~*EMP1*, hsa-miR-126-3p~*CH25H*, hsa-miR-126-3p~*HBB*, hsa-miR-126-3p~*NAMPT*, hsa-miR-126-3p~*POSTN*, and hsa-miR-486-5p~*HBEGF*) and two for non-serous adenocarcinoma (hsa-miR-223-3p~*POSTN* and hsa-miR-223-3p~*PROCR*). One miRNA-mRNA pair was found to be shared between serous and non-serous adenocarcinoma subtypes: hsa-miR-126-3p~*HBEGF*.

**Table 3:** Integrated miRNA and mRNA signatures associated with OS in two subgroups of a cohort of 197 patients: Serous adenocarcinoma (serous) and non-serous adenocarcinoma (NonSerous) compared to 12 miRNA-mRNA interactions identified in 197 patient cohort (All).

Group	Count	Shared miRNA-mRNA pairs
All & Serous & NonSerous	3	hsa-miR-486-5p~*ATF3*
		hsa-miR-486-5p~*HBB*
		hsa-miR-506-3p~*POSTN*
Serous & NonSerous	1	hsa-miR-126-3p~*HBEGF*
All & Serous	9	hsa-miR-126-3p~*PROCR*
		hsa-miR-223-3p~*CH25H*
		hsa-miR-223-3p~*HBEGF*
		hsa-miR-223-3p~*NAMPT*
		hsa-miR-23a-5p~*ATF3*
		hsa-miR-23a-5p~*HBEGF*
		hsa-miR-27a-5p~*ATF3*
		hsa-miR-27a-5p~*EMP1*
		hsa-miR-27a-5p~*HBEGF*
Serous	6	hsa-miR-126-3p~*CH25H*
		hsa-miR-126-3p~*HBB*
		hsa-miR-126-3p~*NAMPT*
		hsa-miR-126-3p~*POSTN*
		hsa-miR-23a-5p~*EMP1*
		hsa-miR-486-5p~*HBEGF*
NonSerous	2	hsa-miR-223-3p~*POSTN*
		hsa-miR-223-3p~*PROCR*

## Discussion

Although many studies have sought to identify molecular signatures for EOC based on miRNA or/and mRNA expression analysis, the results are not consistent. One of the reasons of such discrepancy among various studies may be attributed to the source of miRNAs (whole blood, plasma, or serum) from EOC patients and/or the platform (qPCR or microarray) used for analysis [43]. Moreover, a substantial number of studies on miRNA/mRNA signatures in EOC have been developed based on analyzing tissue from patients with high grade serous OC from The Cancer Genome Atlas (TCGA) database [44–52]. However, the use of only one dataset for discovery may not reflect the heterogeneity of EOC [53]. Additionally, the expression data for 11,864 genes in OC data from TCGA was combined from three different platforms (Agilent, Affymetrix HuEx, Affymetrix U133A) [54]. Although the integrative analysis enables to merge multiple cross-platform datasets into one for identifying biomarkers, the current integration methods have several limitations [55]. Furthermore, most of the prior research has focused on the separate investigation of molecular signatures such as proteins, mRNA or miRNA in relation to various aspects of OC. Here, we explored the interaction between miRNA and mRNA by employing a more stringent approach to (1) assess their expression levels separately, and (2) integrate both analyses on a relevant cohort of EOC patients. 12 miRNA-mRNA interaction pairs related to OS in EOC were identified in this study.

As the full picture of miRNA-mRNA networks has yet to be accomplished [56], the results presented here may be helpful to find key regulators of OC network. In line with our findings, all 6 miRNAs (hsa-miR-126-3p, hsa-miR-223-3p, hsa-miR-23a-5p, hsa-miR-27a-5p, hsa-miR-486-5p, and hsa-miR-506-3p) and 8 mRNAs (*ATF3*, *CH25H*, *EMP1*, *HBB*, *HBEGF*, *NAMPT*, *POSTN*, and *PROCR*) have been previously shown to play an essential role in ovarian carcinogenesis with respect to different aspects such as OS, histological subtype, clinical stage, chemoresistance, or treatment [19, 20, 27, 57–71]. We reported previously that hsa-miR-23a-5p, hsa-miR-27a-5p, and hsa-miR-126-3p, were significantly associated with OS in the same EOC cohort (p < 0.01, univariate Cox regression analysis) [27]. In another study based on miRNA expression profiles from real-time RT-PCR, decreased expression of hsa-miR-486-5p in fresh frozen ovarian serous adenocarcinoma tissues (n = 6) was observed as compared to normal ovary (n = 8) [19]. Hsa-miR-223-3p was observed up-regulated in recurrent versus primary OC tissue, both in fresh frozen and FFPE samples [20]. Moreover, it has been reported that hsa-miR-126-3p presented decreased expression in fresh frozen OC tissues (n = 69) compared with expression in non-cancerous tissue (n = 15) by microarray profiling [57]. However, by RT-PCR analysis, Resnick et al. demonstrated that the same miRNA was elevated in serum from patients diagnosed with EOC (n = 19) when compared to miRNA expression in serum from healthy controls (n = 11) [58].

Hsa-miR-506-3p has emerged as a key network regulator for epithelial-to-mesenchymal transition, which is one of the initiating steps of epithelial tumors metastasis [13, 48, 59]. The expression level of hsa-miR-506-3p in fresh frozen tissues was substantially decreased in 20 primary OC tissues (n = 20 each) compared to the normal tissues (n = 20) (p<0.001) [60]. Bagnoli et al. identified a panel of 35 miRNA predictors of risk of OC relapse or progression, including hsa-miR-506-3p [61]. Additionally, hsa-miR-506-3p and *CH25H* have been reported to be a part of recurrence-associated multi-RNA signature to predict disease-free survival in data from a cohort of 322 OC patients from TCGA [62]. Through the validation of OC data from TCGA database, *POSTN* has been found to be one of the five hub genes linked with poor prognosis [63]. Kujawa et al. proposed the joint *POSTN* and *FN1* scored as an independent prognostic factor for OS in OC based on immunohistochemical analysis of 108 FFPE tissues from patients with last stage OC who did not receive neoadjuvant chemotherapy [64]. In another study, high expression of *HBB* has been proposed as a predictor for a shorter 5-year survival and has been shown to be associated with drug resistance in OC based on the microarray data from different resources: TCGA Ovarian Statistics, Bonome Ovarian Statistics, Yoshihara Ovarian Statistics, Lu Ovarian Statistics and Welsh Ovarian Statistics [65]. Liu et al. presented that epithelial membrane protein 1 mRNA (*EMP1*) has been up-regulated in 34 OC tissues samples when compared with corresponding noncancerous ovarian epithelial tissues [66]. Moreover, in the same study, *EMP1* expression has been linked with clinical classification, metastasis, and survival prognosis in OC. Vert et al. demonstrated that activating transcription factor 3 (*ATF3*) in OC cell lines has a crucial role for antitumor activity and to strengthen the antiviral properties of an RNA-damaging drug, Onconase [67]. The inhibition of heparin-binding epidermal growth factor-like growth factor (*HBEGF*) has been proposed as a novel therapeutic strategy for patients with paclitaxel‐resistant OC [68, 69]. Overexpression of nicotinamide phosphoribosyltransferase (*NAMPT*) has been described across a broad range of solid tumors including ovarian, colorectal, breast, gastric, prostate, and endometrial carcinomas in addition with melanoma, gliomas, and astrocytomas indicating that inhibition of *NAMPT* may be used as a cancer treatment strategy [70]. It has been reported that the increase in the levels of endothelial protein C receptor (*PROCR*, also known as *EPCR*) in the plasma of OC patients, parallels the increase in CA125 [71].

The mechanism by which miRNAs target specific genes is poorly understood and to find more information about the role of a particular miRNA, their physical binding sites need to be determined [15]. We found 1 negatively and 11 positively correlated miRNA-mRNA interaction pairs between 6 miRNAs and 8 mRNAs (Table 2). It was primarily acknowledged that miRNAs act as negative regulators of gene expression either by conducting the degradation or blocking the translation of their targets’ mRNAs [6]. However, many studies have shown the co-existence of negative and positive miRNA-mRNA correlations, suggesting that miRNAs function as modulators of miRNA-mRNA interactions rather than as only on-off molecular switches [21, 72, 73]. Tan et al. explored the positive correlation between miRNAs and mRNAs across 31 major human cancers and found that many of these correlations are prevalent and consistent across cancer types [21]. Among significantly positive miRNA-mRNA pairs correlation covering at least 10 cancer types, 2 pairs from our list can be found: hsa-miR-486-5p~*HBB* and hsa-miR-223-3p~*HBEGF*. The calculated Pearson’s correlation coefficients were similar between Tan et al.’s and our studies: 0.70 and 0.74 for hsa-miR-486-5p~*HBB*, as well as 0.34 and 0.42 for hsa-miR-223-3p~*HBEGF*. Most of the positive correlations (~87%) reported in the Tan et al.’s study, including hsa-miR-223-3p~*HBEGF*, could be explained by various mechanisms, such as the miRNA-host gene co-expression, inhibition of the upstream suppressor of the gene, co-transcription by shared transcription factors, enhancer-mediated miRNA-gene co-expression or direct binding of miRNA to the gene promoters [21].

Furthermore, Jacobsen et al. developed an algorithm to evaluate the association between miRNA and mRNA expression in the presence of DNA copy-number and promoter methylation aberrations [42]. Interestingly, based on that algorithm, four miRNA-mRNA signatures from our study showed very high association rank values in OC: hsa-miR-506-3p~*POSTN* (rank 1 out of 1942), hsa-miR-486-5p~*HBB* (rank 2 out of 1685), hsa-miR-27a-5p~*ATF3* (rank 8 out of 1658), and hsa-miR-486-5p~*ATF3* (rank 23 out of 1685) (Table 2). These findings indicate that our study has a potential to identify relevant miRNA-mRNA interaction pairs in EOC. Some limitations should be noted. First, the identified miRNA-mRNA interaction pairs related to OC are based on statistical evidence and they require further experimental validation. Unfortunately, we could not find any public dataset with both miRNA and mRNA studies run on the Affymetrix platform, as it has been done in our publication. We believe that the strength of our study lies in the fact that global miRNA and mRNA profiling was done in the same cohort eliminating different analytical runs, material quality etc. Furthermore, to our knowledge, the current work presents for the first time an integrated analysis between miRNA and mRNA in EOC patients by employing the same platform and analysis pipeline, and not using The Cancer Genome Atlas (TCGA) dataset. Second, we investigated the relationship between miRNAs and mRNAs, by using expression data. However, mRNA expression levels do not necessarily reflect protein expression, as protein translation may be regulated in various ways. Therefore, in the next step, integrative analysis of miRNAs, mRNAs and protein expression data could provide further basis for potential future application [74].

We have further investigated whether the overrepresentation of serous adenocarcinoma subtype in our cohort skewed our observations. All twelve miRNA-mRNA interactions identified in the whole cohort were among nineteen pairs found in the serous adenocarcinoma subgroup. Three miRNA-mRNA signature pairs were shared by the whole cohort and two subgroups: serous and non-serous adenocarcinoma. However, there were also some differences observed between compared groups. These results indicate that the overrepresentation of one subtype indeed affected the results. However, as specific miRNA-mRNA signatures were discovered for both subtypes: 6 for serous adenocarcinoma and 2 for non-serous adenocarcinoma, our study might be useful to reveal miRNA-mRNA signatures with a prognostic value for OS in different subtypes of EOC. The content of our cohort reflects a real-world scenario of EOC, comprising five main subtypes: high-grade serous (70%), endometrioid (10%), clear cell (10%), mucinous (3%), and low-grade serous (<5%) [8]. Nevertheless, for discovery of specific miRNA-mRNA interaction pairs in each histologic type, further studies should expand on such cohorts to provide larger sample sizes for each subtype.

In conclusion, the miRNA-mRNA signatures identified in this study may serve as promising candidates for subsequent *in vitro* validation to understand the role of miRNAs on the expression of the mRNAs in EOC and reveal pairs with a prognostic value for OS in different subtypes of EOC.

## Supporting information

S1 Fig Analysis pipeline for discovery of miRNA and mRNA interactions. (PDF)

S2 Fig Correlation plots for 12 miRNA-mRNA signatures associated with OS in 197 OC cohort. (PDF)

S3 Fig Kaplan-Meier curves of patients with and without the miRNA-mRNA signatures. (PDF)

S1 Table Multivariate analysis of OS with following clinical features included in the model: Stage (early vs late), type (I vs II), treatment response (>60 months before progression of disease vs <60 months before progression of disease), menopause (pre-menopausal vs post-menopausal). (XLSX)

S2 Table 17 miRNAs and 30 mRNAs targets selected for pearson correlation test to identify integrated microRNA and mRNA signatures associated with overall survival in EOC–literature study. (DOC)
