# Correction: Integrated microRNA and mRNA signatures associated with overall survival in epithelial ovarian cancer

**DOI:** 10.1371/journal.pone.0315859

**Published:** 2024-12-11

**Authors:** Joanna Lopacinska-Jørgensen, Douglas V. N. P. Oliveira, Guy Wayne Novotny, Claus K. Høgdall, Estrid V. Høgdall

The third author’s initials appear incorrectly in the citation. The correct citation is: Lopacinska-Jørgensen J, Oliveira DVNP, Novotny GW, Høgdall CK, Høgdall EV (2021) Integrated microRNA and mRNA signatures associated with overall survival in epithelial ovarian cancer. PLOS ONE 16(7): e0255142. https://doi.org/10.1371/journal.pone.0255142.
