# Supplementary figures and images for: Integrated microRNA and mRNA signatures associated with overall survival in epithelial ovarian cancer
Source: PLoS One. 2021 Jul 28;16(7):e0255142. doi: 10.1371/journal.pone.0255142 (PMC8318284; doi:10.1371/journal.pone.0255142)

# Analysis pipeline for discovery of miRNA and mRNA interactions

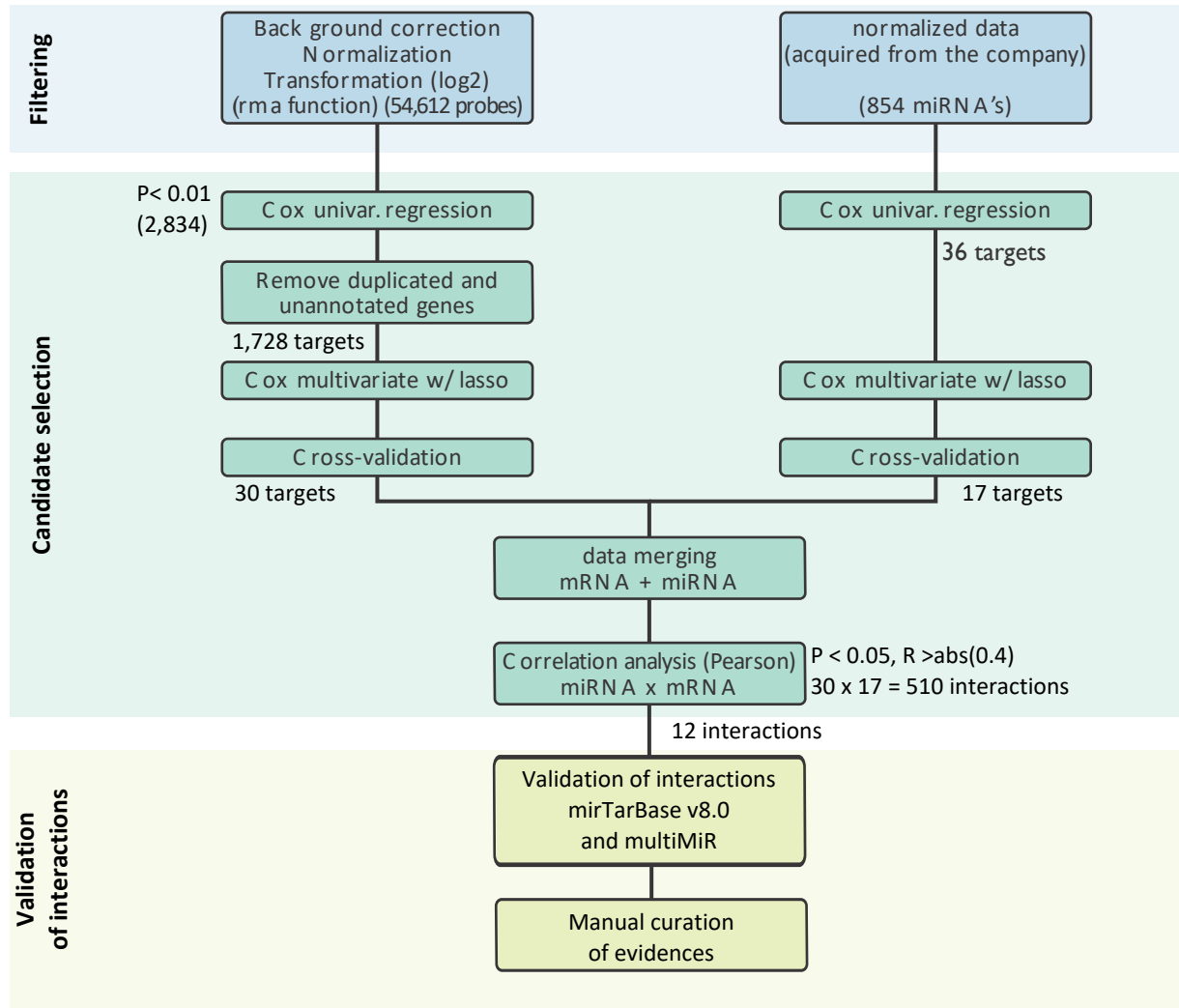

Supplement: S1 Fig — (PDF) [file pone.0255142.s001.pdf]

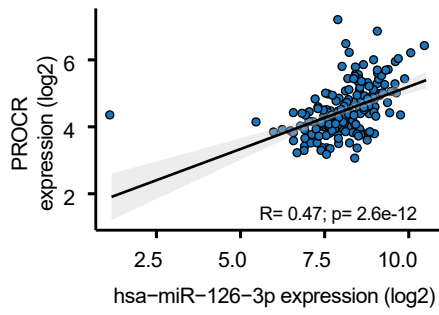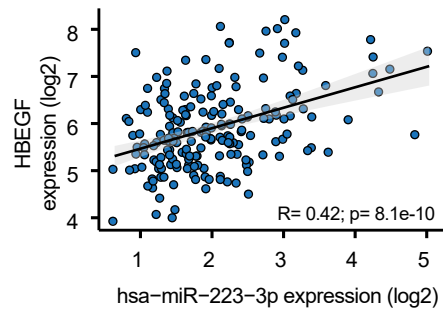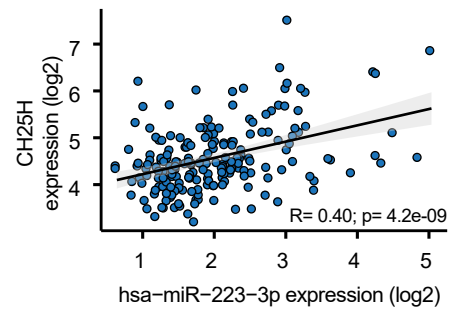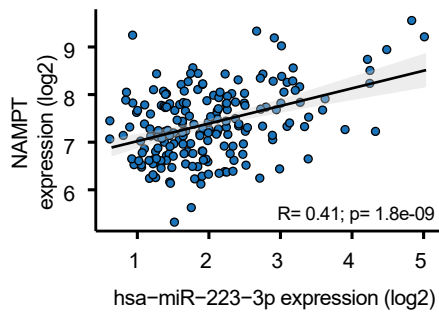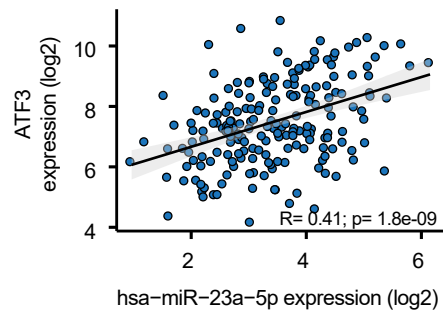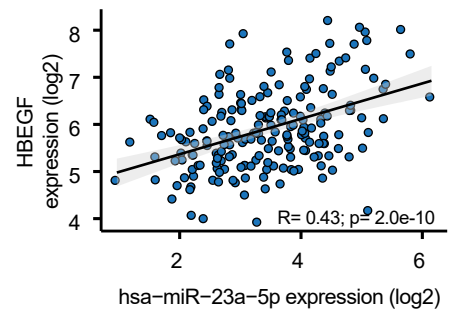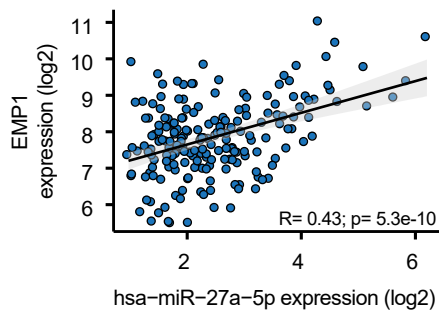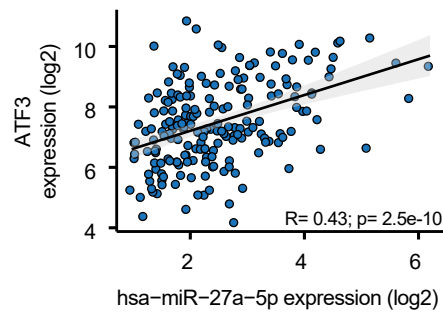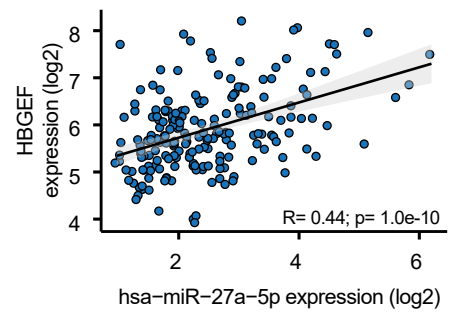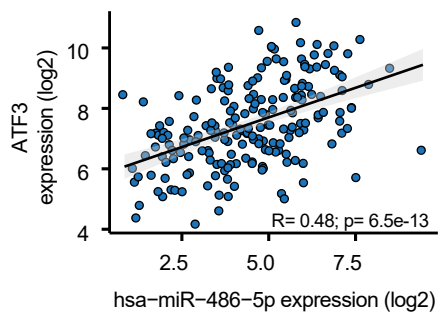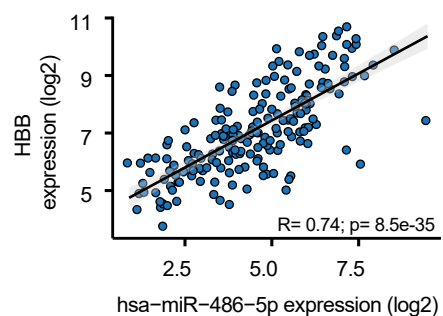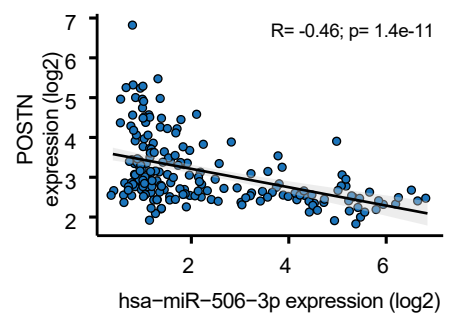

Supplement: S2 Fig — (PDF) [file pone.0255142.s002.pdf]
