# Supplementary material for: Integrated microRNA and mRNA signatures associated with overall survival in epithelial ovarian cancer
Source: PLoS One. 2021 Jul 28;16(7):e0255142. doi: 10.1371/journal.pone.0255142 (PMC8318284; doi:10.1371/journal.pone.0255142)

Candidate pair: hsa-miR-126-3p ~ *PROCR*

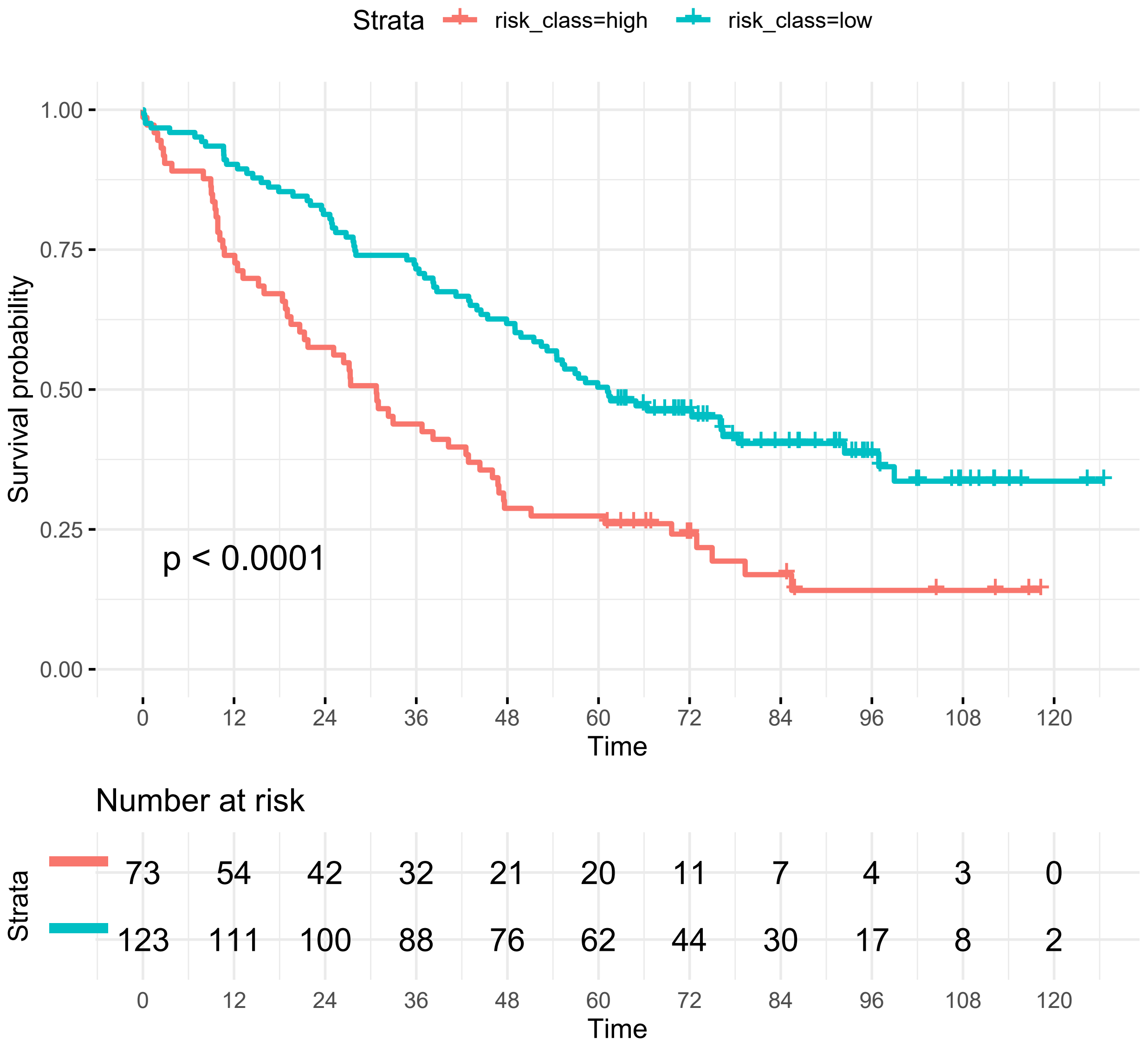

Candidate pair: hsa-miR-223-3p ~ *HBEGF*

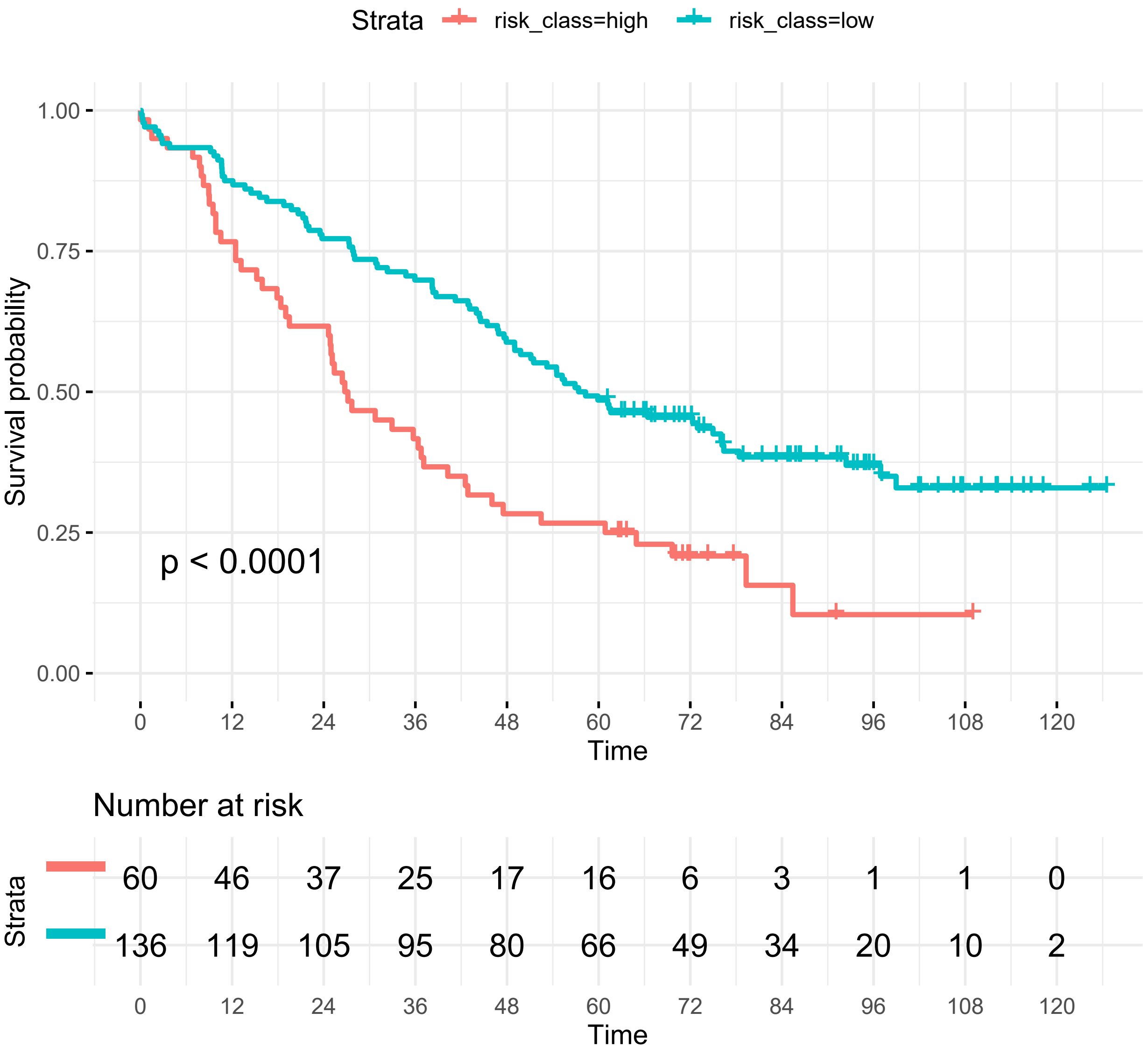

Candidate pair: hsa-miR-223-3p ~ *CH25H*

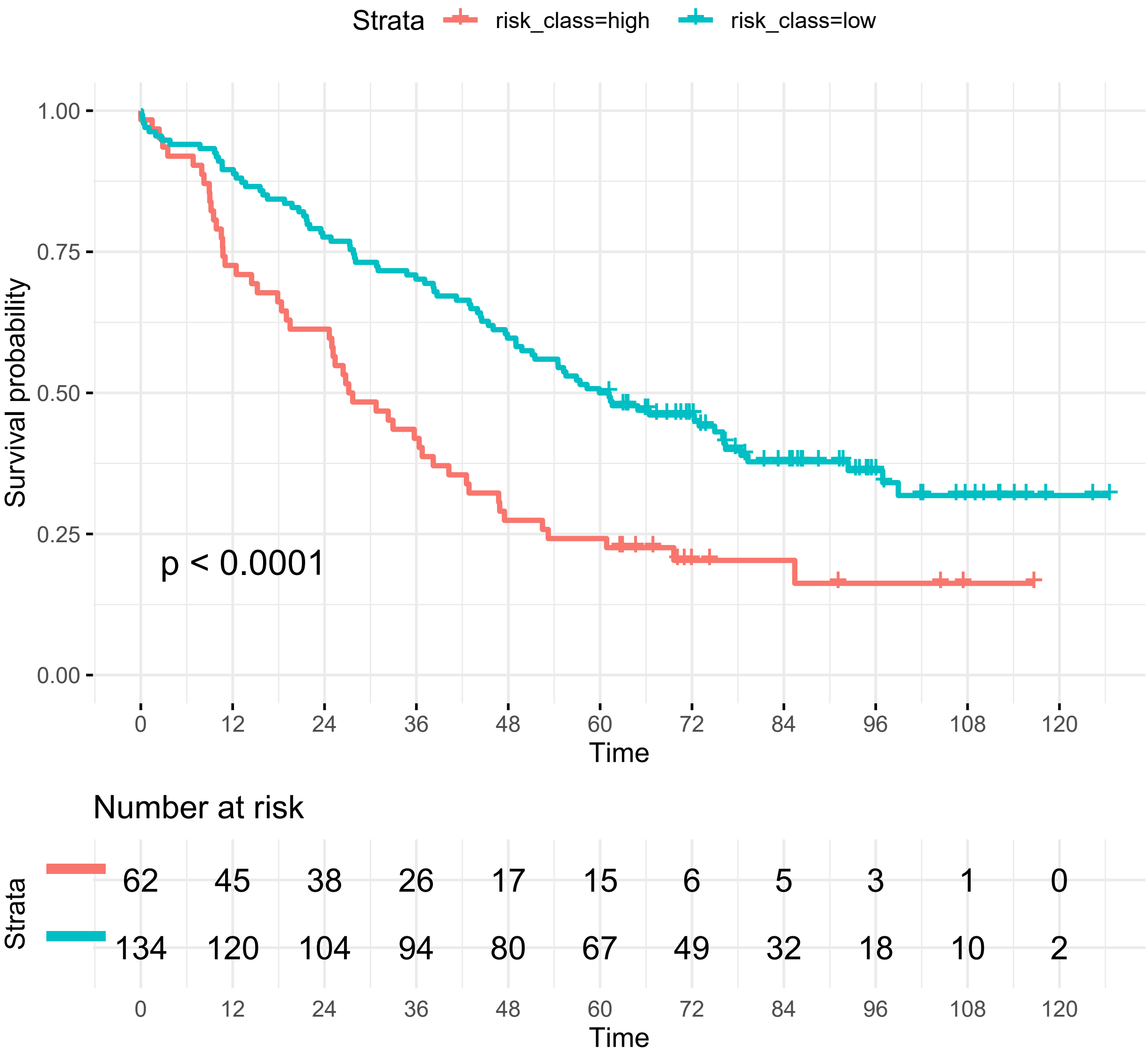

Candidate pair: hsa-miR-223-3p ~ *NAMPT*

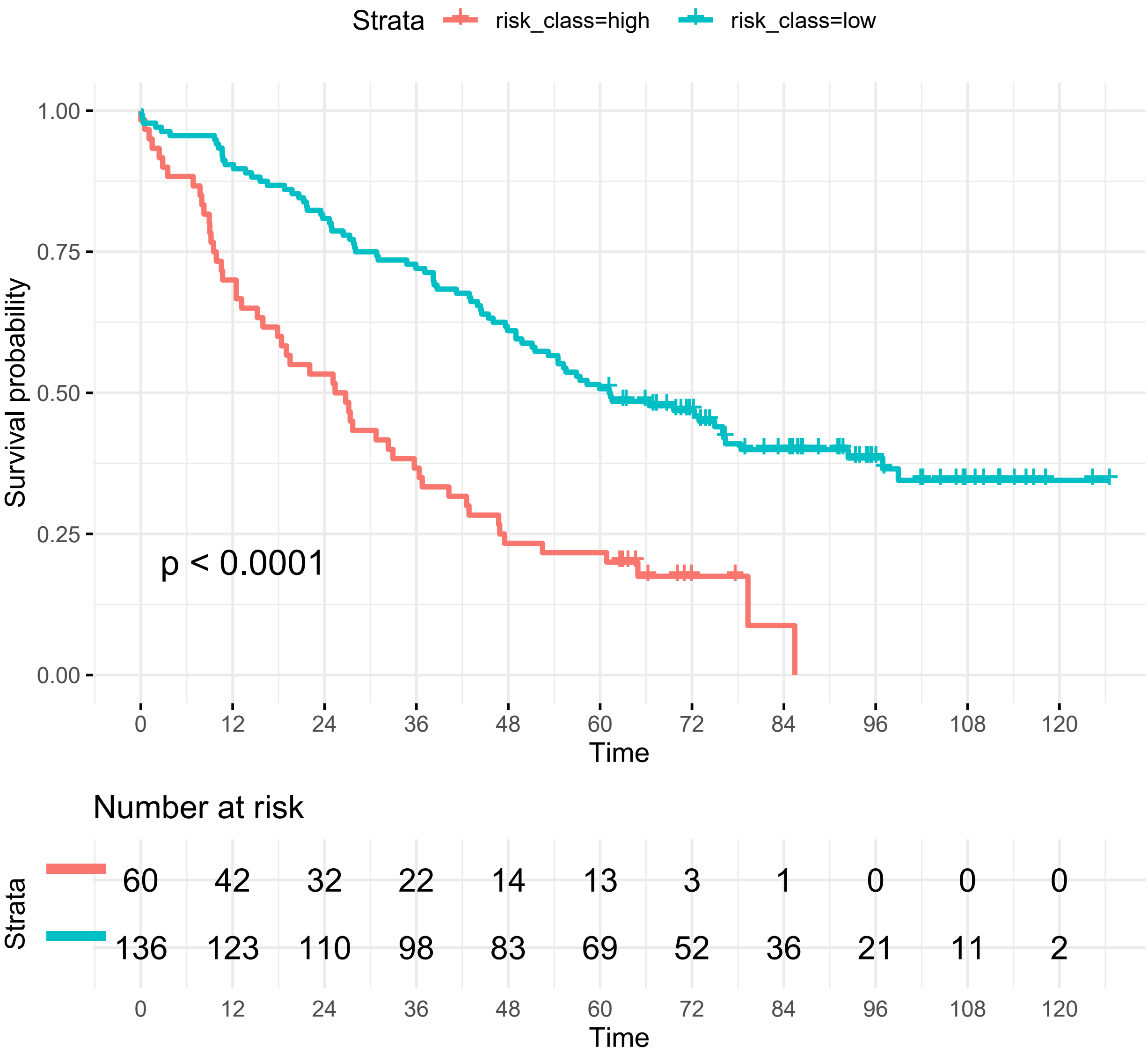

Candidate pair: hsa-miR-23a-5p ~ *ATF3*

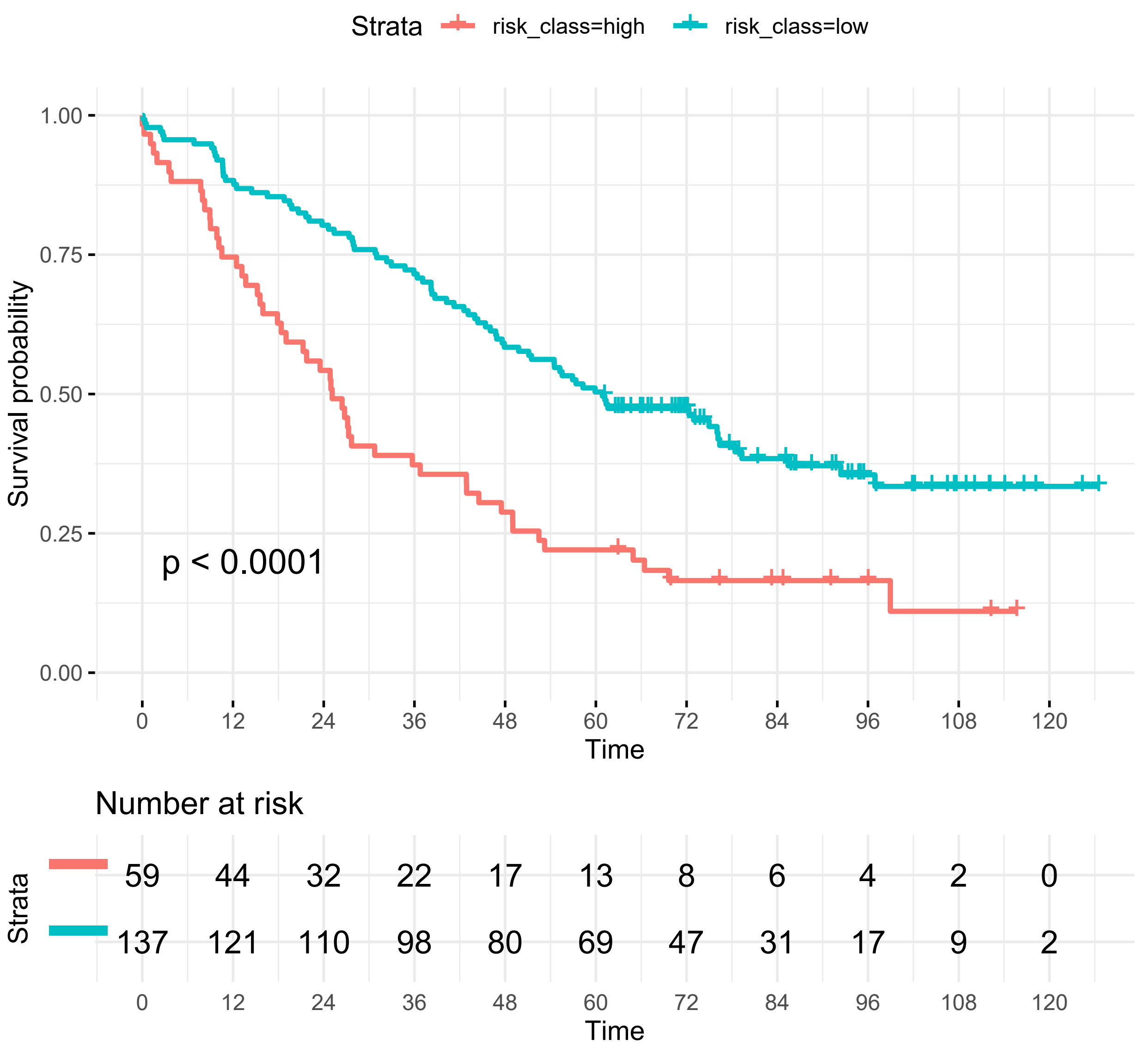

Candidate pair: hsa-miR-23a-5p ~ *HBEGF*

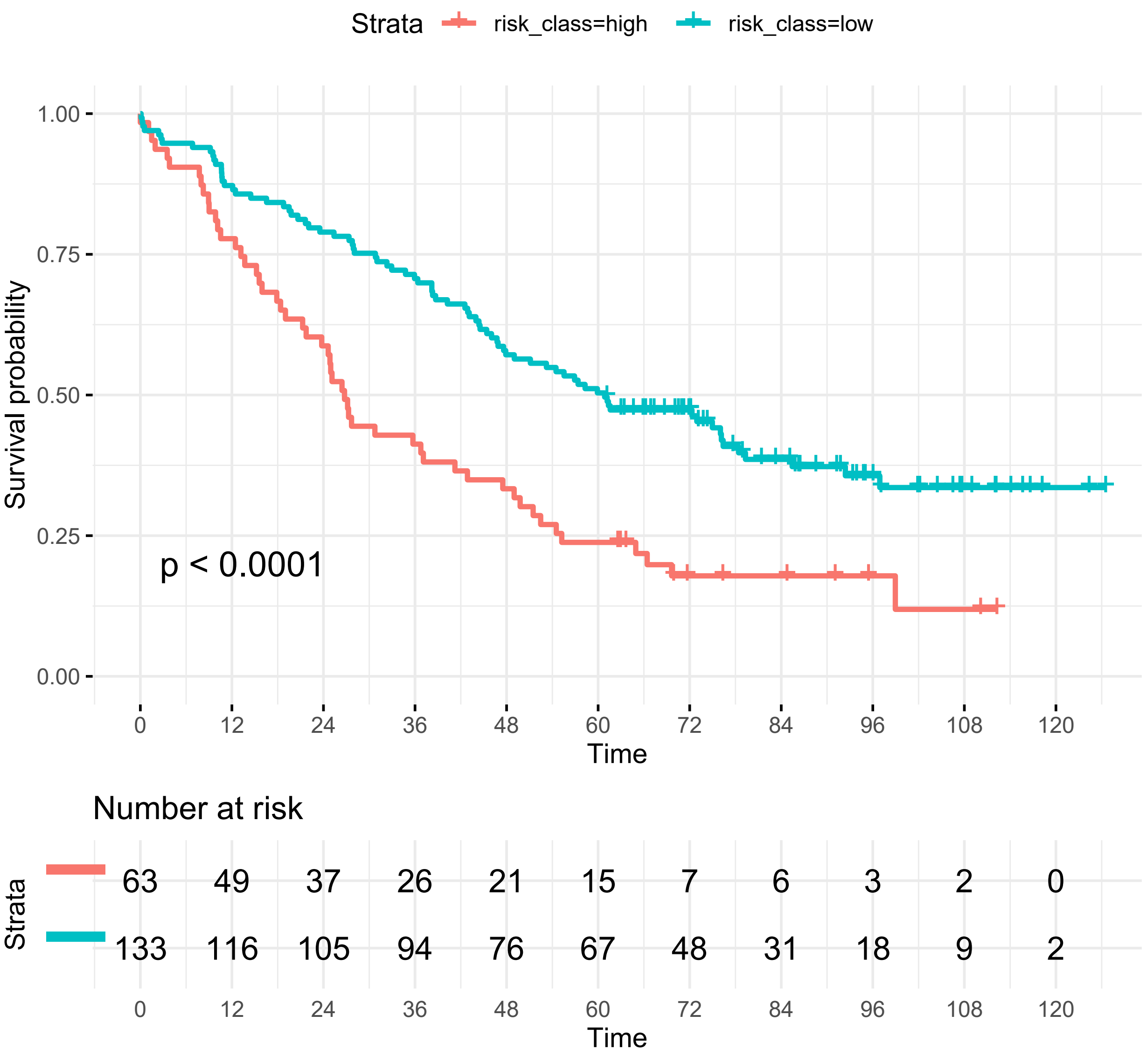

Candidate pair: hsa-miR-27a-5 p ~ *EMP1*

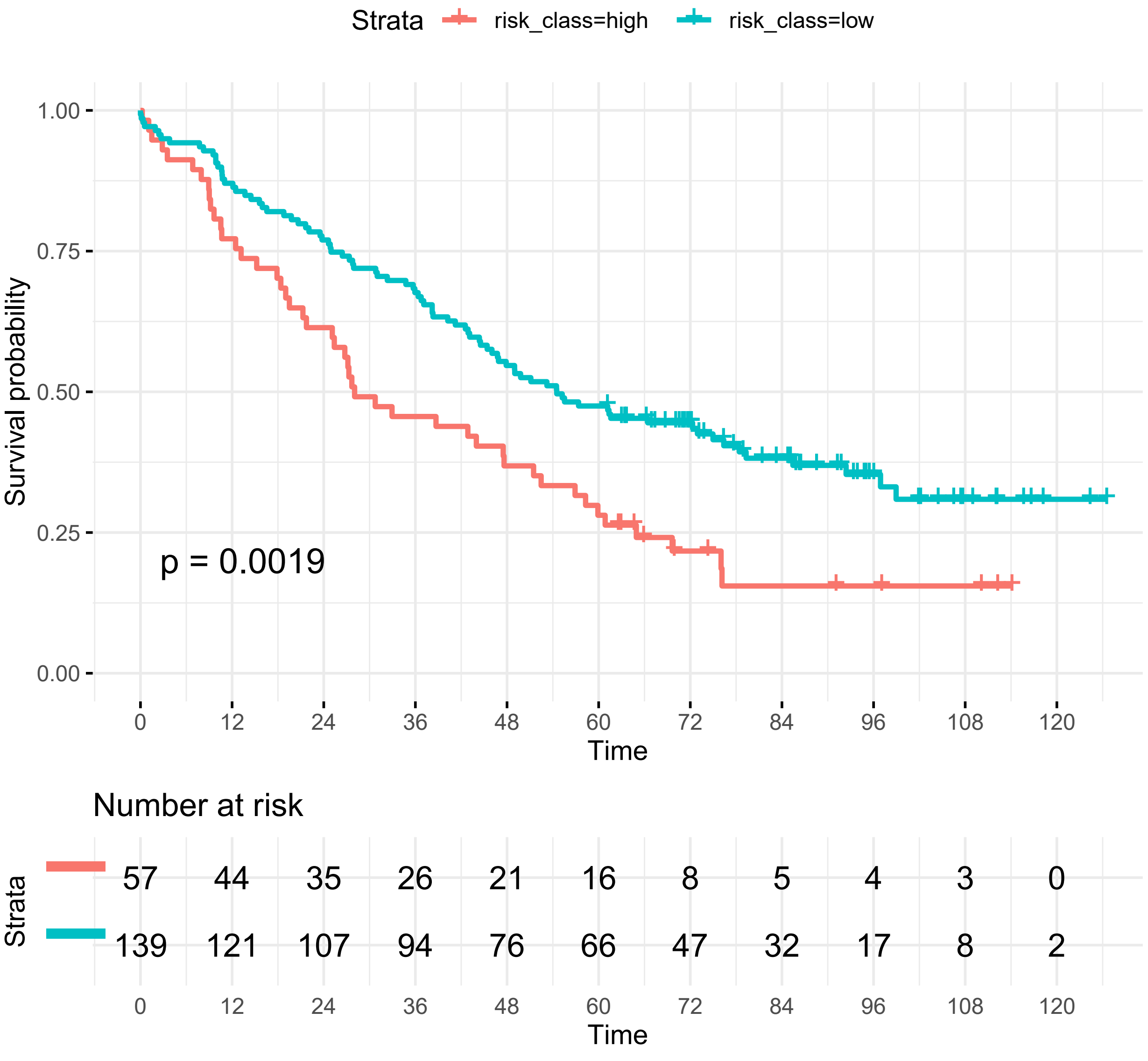

Candidate pair: hsa-miR-27a-5p ~ *ATF3*

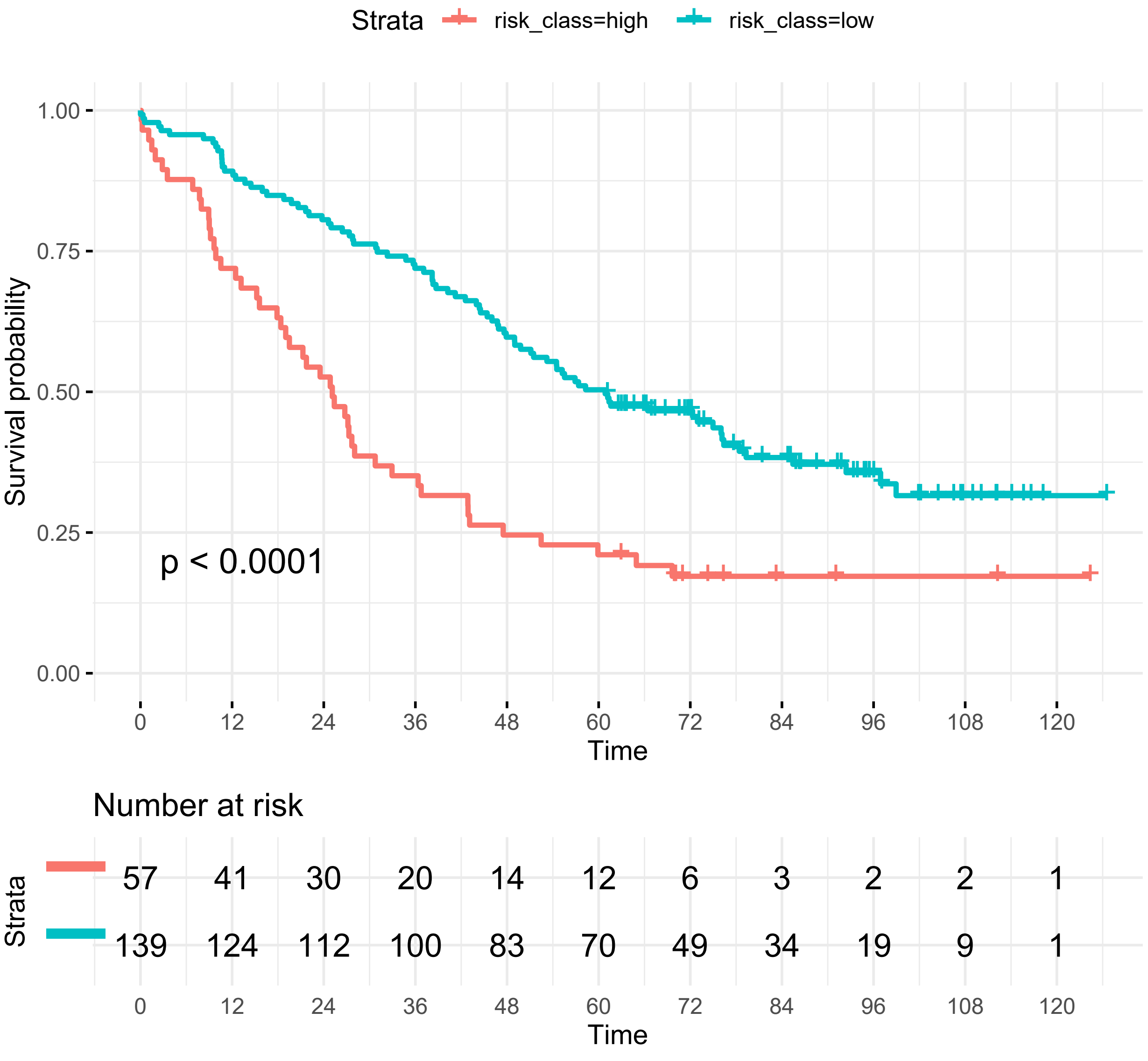

Candidate pair: hsa-miR-27a-5p ~ *HBEGF*

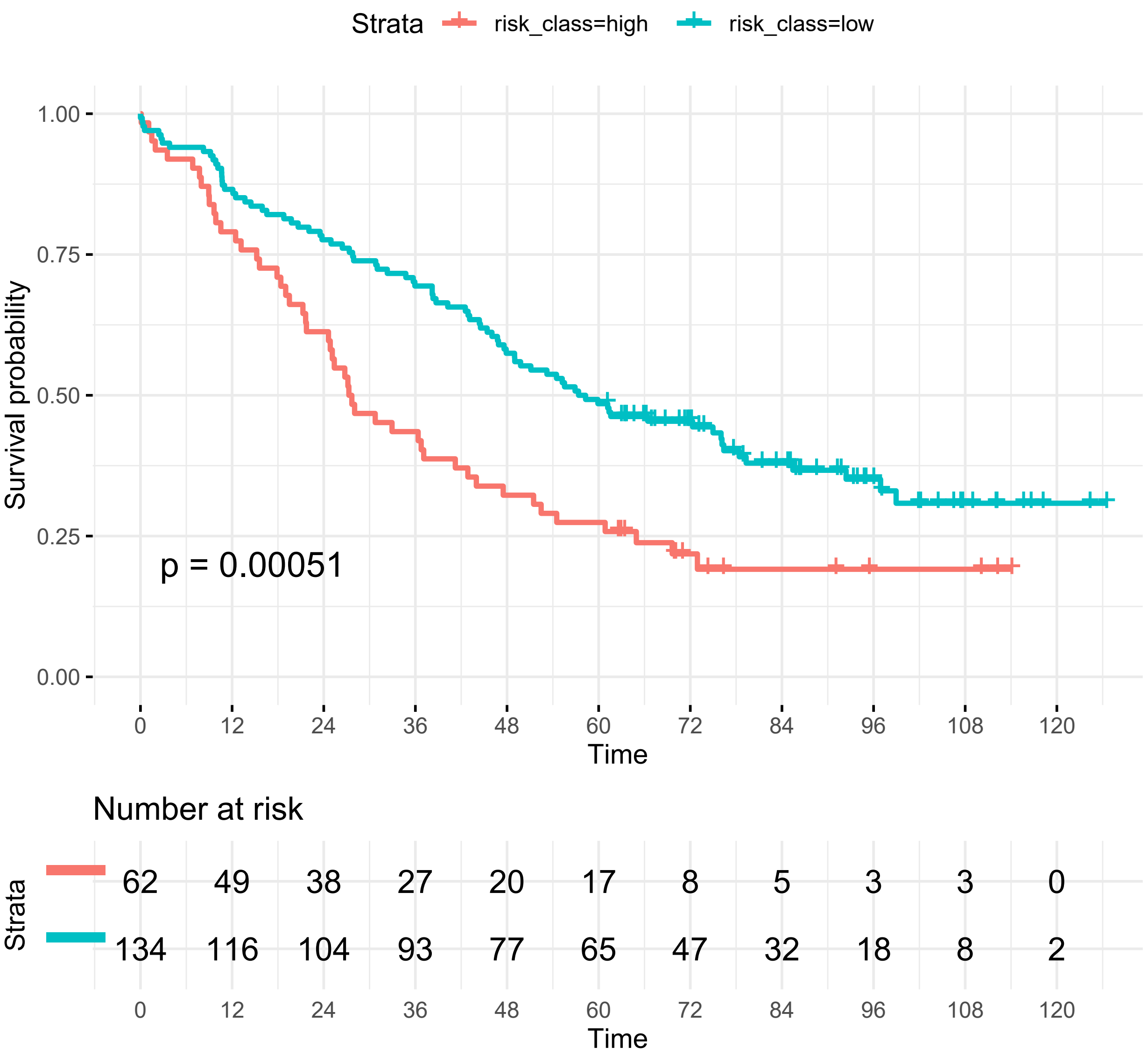

Candidate pair: hsa-miR-486-5p ~ *ATF3*

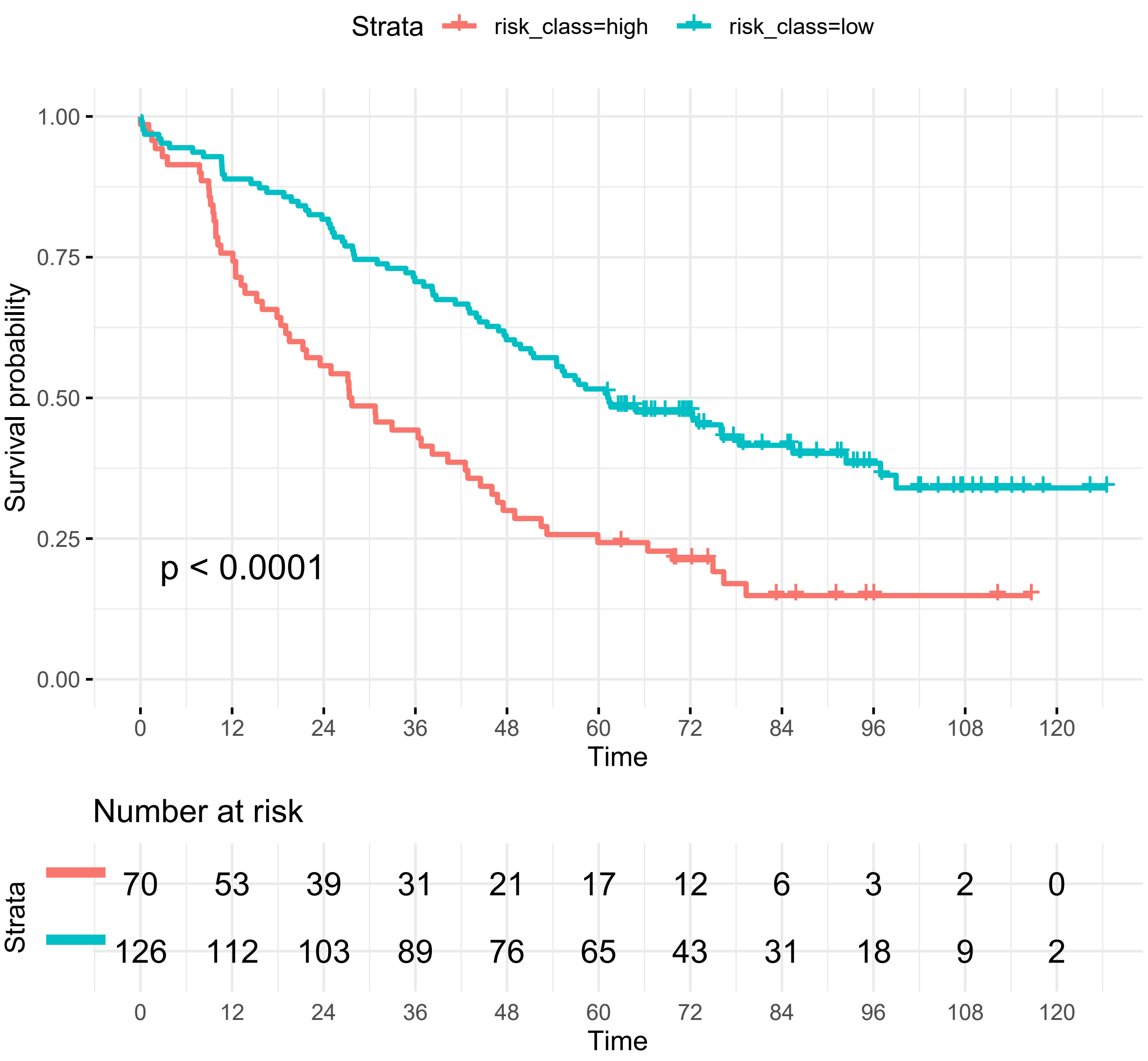

Candidate pair: hsa-miR-486-5p ~ *HBB*

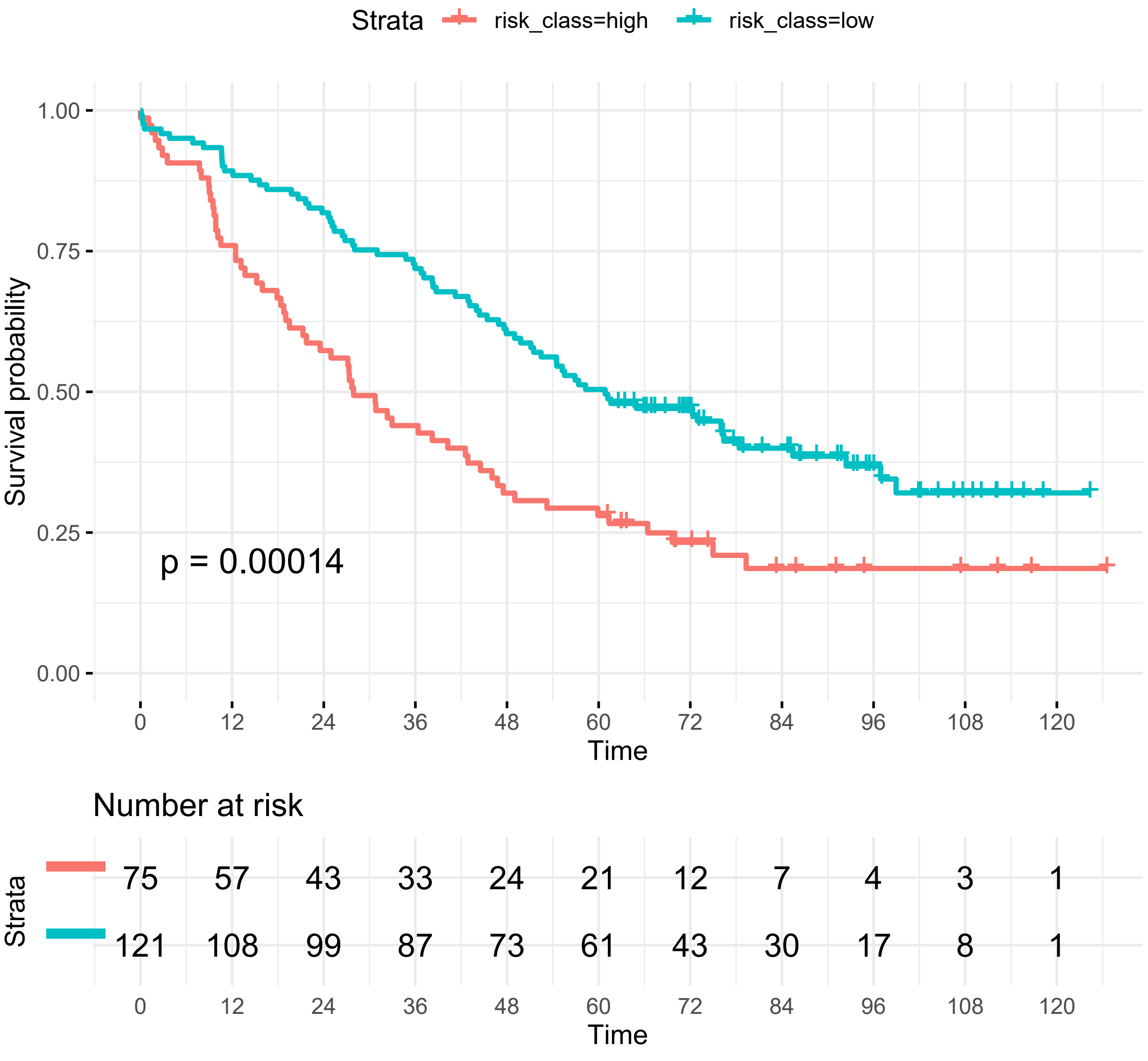

Candidate pair: hsa-miR-506-3p ~ *POSTN*

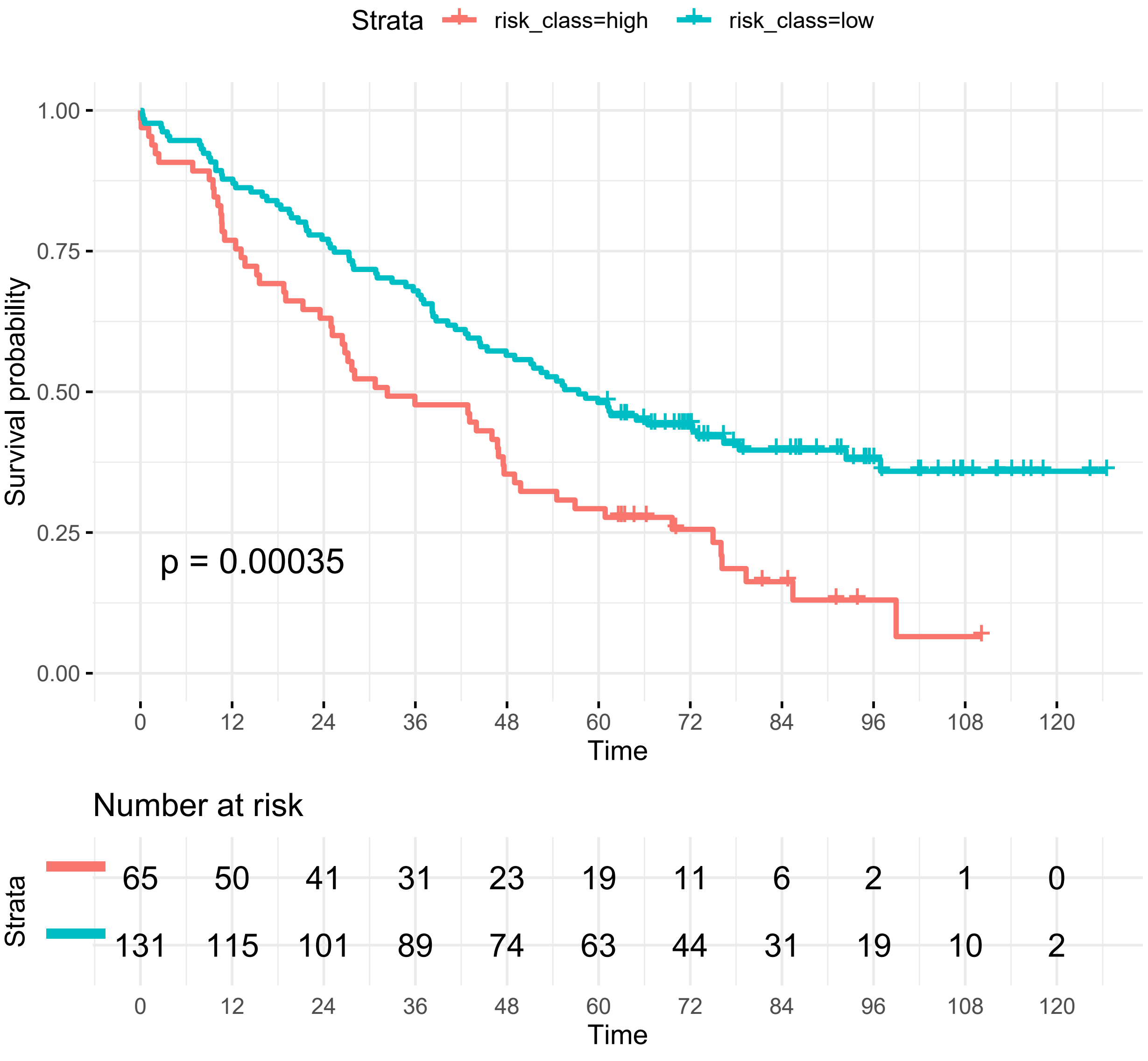

Supplement: S3 Fig — (PDF) [file pone.0255142.s003.pdf]
