# Supplementary material for: Integrated microRNA and mRNA signatures associated with overall survival in epithelial ovarian cancer
Source: PLoS One. 2021 Jul 28;16(7):e0255142. doi: 10.1371/journal.pone.0255142 (PMC8318284; doi:10.1371/journal.pone.0255142)
Supplement: S2 Table — (DOC) [file pone.0255142.s005.doc]

**S2 Table. 17 miRNAs and 30 mRNAs targets selected for Pearson correlation test to identify integrated microRNA and mRNA signatures associated with overall survival in EOC – literature study. A search was performed in the PubMed database, using the keywords: “miRNA or mRNA specific name” and “ovarian/ovary”. If there were no records related to ovarian cancer, a search was conducted for “cancer”, e.g. (miR-576-5p[Title/Abstract]) AND (cancer[Title/Abstract]).**

|  | |
| --- | --- |
| miRNA/mRNA | References |
| hsa-miR-1183 | Identification and validation of potential prognostic and predictive miRNAs of epithelial ovarian cancer (1) |
| hsa-miR-125a-3p | HULC functions as an oncogene in ovarian carcinoma cells by negatively modulating miR-125a-3p (2)  MicroRNA expression profiles in serous ovarian carcinoma (3) |
| hsa-miR-126-3p | MiR-126-3p inhibits ovarian cancer proliferation and invasion via targeting PLXNB2 (4)  Identification and validation of potential prognostic and predictive miRNAs of epithelial ovarian cancer (1) |
| hsa-miR-198 | Circ0004390 promotes cell proliferation through sponging miR-198 in ovarian cancer (5) |
| hsa-miR-223-3p | MicroRNA-223-3p regulates ovarian cancer cell proliferation and invasion by targeting SOX11 expression (6)  miR-223 potentially targets SWI/SNF complex protein SMARCD1 in atypical proliferative serous tumor and high-grade ovarian serous carcinoma (7)  Potential role of miR-9 and miR-223 in recurrent ovarian cancer (8) |
| hsa-miR-23a-5p | Identification and validation of potential prognostic and predictive miRNAs of epithelial ovarian cancer (1) |
| hsa-miR-23a-3p | Identification and validation of potential prognostic and predictive miRNAs of epithelial ovarian cancer (1)  Correlation analysis on the expression levels of microRNA-23a and microRNA-23b and the incidence and prognosis of ovarian cancer (9) |
| hsa-miR-27a-5p | Identification and validation of potential prognostic and predictive miRNAs of epithelial ovarian cancer (1) |
| hsa-miR-486-5p | Estrogen receptor-mediated miR-486-5p regulation of OLFM4 expression in ovarian cancer (10) |
| hsa-miR-506-3p | Microrna-506-3p inhibits proliferation and promotes apoptosis in ovarian cancer cell via targeting sirt1/akt/foxo3a signaling pathway (11)  Recurrence-associated multi-RNA signature to predict disease-free survival for ovarian cancer patients (12) |
| hsa-miR-513a-3p | MiR-513a-3p inhibits EMT mediated by HOXB7 and promotes sensitivity to cisplatin in ovarian cancer cells (13) |
| hsa-miR-576-5p | 11 PubMed cancer-related publications ((miR-576-5p[Title/Abstract]) AND (cancer[Title/Abstract])) |
| hsa-miR-586 | 2 PubMed cancer-related publications ((miR-586[Title/Abstract]) AND (cancer[Title/Abstract])) |
| hsa-miR-615-5p | 14 PubMed cancer-related publications ((miR-619-5p[Title/Abstract]) AND (cancer[Title/Abstract])) |
| hsa-miR-619-3p | - |
| hsa-miR-665 | MicroRNA‑665 suppresses the growth and migration of ovarian cancer cells by targeting HOXA10 (14)  MicroRNA-665 promotes the proliferation of ovarian cancer cells by targeting SRCIN1 (15) |
| hsa-miR-876-3p | 10 PubMed publications cancer-related publications ((miR-876-3p[Title/Abstract]) AND (cancer[Title/Abstract])) |
| *ADGRL1* | 1 PubMed cancer-related publication ((ADGRL1[Title/Abstract]) AND (cancer[Title/Abstract])) |
| *ANKRD27* | - |
| *APOBEC3C* | 14 PubMed cancer-related publications ((APOBEC3C[Title/Abstract]) AND (cancer[Title/Abstract])) |
| *APOLD1* | 3 PubMed cancer-related publications ((APOLD1[Title/Abstract]) AND (cancer[Title/Abstract])) |
| *ATF3* | Activating transcription factor 3 is crucial for antitumor activity and to strengthen the antiviral properties of Onconase (16) |
| *BTN3A2* | BTN3A2 serves as a prognostic marker and favors immune infiltration in triple-negative breast cancer (17) |
| *CCDC102B* | - |
| *CCDC57* | 1 PubMed cancer-related publication ((CCDC57[Title/Abstract]) AND (cancer[Title/Abstract])) |
| *CCL5* | Ovarian cancer stem cells promote tumour immune privilege and invasion via CCL5 and regulatory T cells (18)  CCL2/CCL5 secreted by the stroma induce IL-6/PYK2 dependent chemoresistance in ovarian cancer (19) |
| *CD99* | Nrf2 induced cisplatin resistance in ovarian cancer by promoting CD99 expression (20) |
| *CH25H* | Recurrence-associated multi-RNA signature to predict disease-free survival for ovarian cancer patients (12) |
| *EMP1* | EMP1 promotes the proliferation and invasion of ovarian cancer cells through activating the MAPK pathway (21) |
| *FOXD1* | FOXD1 is targeted by miR-30a-5p and miR-200a-5p and suppresses the proliferation of human ovarian carcinoma cells by promoting p21 expression in a p53-independent manner (22) |
| *FPR1* | Screening of Critical Genes Involved in Metastasis and Prognosis of High-Grade Serous Ovarian Cancer by Gene Expression Profile Data (23)  An IL6-correlated signature in serous epithelial ovarian cancer associates with growth factor response (24) |
| *HBB* | Discovery of microarray-identified genes associated with ovarian cancer progression (25) |
| *HBEGF* | CRM197 reverses paclitaxel resistance by inhibiting the NAC-1/Gadd45 pathway in paclitaxel-resistant ovarian cancer cells (26)  Discovery of HB-EGF binding peptides and their functional characterization in ovarian cancer cell lines (27) |
| *HPS1* | - |
| *HSPA1A* | HSPA1A, HSPA1L and TRAP1 heat shock genes may be associated with prognosis in ovarian epithelial cancer (28)  Secretion of cytokines and heat shock protein (HspA1A) by ovarian cancer cells depending on the tumor type and stage of disease (29) |
| *KDM8* | 8 PubMed publications cancer-related publications ((KDM8[Title/Abstract]) AND (cancer[Title/Abstract])) |
| *L1CAM* | Clinical impact of L1CAM expression measured on the transcriptome level in ovarian cancer (30)  Prognostic significance of L1CAM in ovarian cancer and its role in constitutive NF-κB activation (31) |
| *NAMPT* | Nicotinamide phosphoribosyltransferase in malignancy: a review (32) |
| *OPA1* | p53 is required for cisplatin-induced processing of the mitochondrial fusion protein L-Opa1 that is mediated by the mitochondrial metallopeptidase Oma1 in gynecologic cancers (33) |
| *PDAP1* | 2 PubMed cancer-related publications ((PDAP1[Title/Abstract]) AND (cancer[Title/Abstract])) |
| *PGR* | PGR +331 A/G and increased risk of epithelial ovarian cancer (34) |
| *POSTN* | Systematic prediction of key genes for ovarian cancer by co-expression network analysis (35)  Fibronectin and periostin as prognostic markers in ovarian cancer (36) |
| *PROCR* | Endothelial protein C receptor expressed by ovarian cancer cells as a possible biomarker of cancer onset |
| *RAB3A* | 12 PubMed cancer-related publications ((RAB3A[Title/Abstract]) AND (cancer[Title/Abstract])) |
| *STX18* | 3 PubMed cancer-related publications ((STX18[Title/Abstract]) AND (cancer[Title/Abstract])) |
| *TANK* | 75 PubMed cancer-related publications ((tank[Title/Abstract]) AND ((kinase[Title/Abstract]) AND (cancer[Title/Abstract])) |
| *TUBB6* | Methylomic Analysis of Ovarian Cancers Identifies Tumor-Specific Alterations Readily Detectable in Early Precursor Lesions (37) |

1. Philipsen K, Id P, Claus H, Karlsen MA, Christensen J, Novotny GW, et al. Identification and validation of potential prognostic and predictive miRNAs of epithelial ovarian cancer. PLoS One. 2018;13(11):1–18.

2. Chu P, Xu L, Su H. HULC functions as an oncogene in ovarian carcinoma cells by negatively modulating miR-125a-3p. J Physiol Biochem. 2019;75(2):163–71.

3. Nam EJ, Yoon H, Kim SW, Kim H, Kim YT, Kim JH, et al. MicroRNA expression profiles in serous ovarian carcinoma. Clin Cancer Res. 2008;14(9):2690–5.

4. Xiang G, Cheng Y. MiR-126-3p inhibits ovarian cancer proliferation and invasion via targeting PLXNB2. Reprod Biol. 2018;18(3):218–24.

5. Xu F, Ni M, Li J, Cheng J, Zhao H, Zhao J, et al. Circ0004390 promotes cell proliferation through sponging miR-198 in ovarian cancer. Biochem Biophys Res Commun. 2020 May 21;526(1):14–20.

6. Fang G, Liu J, Wang Q, Huang X, Yang R, Pang Y, et al. MicroRNA-223-3p regulates ovarian cancer cell proliferation and invasion by targeting SOX11 expression. Int J Mol Sci. 2017;18(6):1208.

7. Arts FA, Keogh L, Smyth P, O’Toole S, Ta R, Gleeson N, et al. miR-223 potentially targets SWI/SNF complex protein SMARCD1 in atypical proliferative serous tumor and high-grade ovarian serous carcinoma. Hum Pathol. 2017 Dec 1;70:98–104.

8. Laios A, O’Toole S, Flavin R, Martin C, Kelly L, Ring M, et al. Potential role of miR-9 and miR-223 in recurrent ovarian cancer. Mol Cancer. 2008;7:1–14.

9. Su L, Liu M. Correlation analysis on the expression levels of microRNA-23a and microRNA-23b and the incidence and prognosis of ovarian cancer. Oncol Lett. 2018;16(1):262–6.

10. Ma H, Tian T, Liang S, Liu X, Shen H, Xia M, et al. Estrogen receptor-mediated miR-486-5p regulation of OLFM4 expression in ovarian cancer. Oncotarget. 2016;7(9):10594–605.

11. Xia XY, Yu YJ, Ye F, Peng GY, Li YJ, Zhou XM. Microrna-506-3p inhibits proliferation and promotes apoptosis in ovarian cancer cell via targeting sirt1/akt/foxo3a signaling pathway. Neoplasma. 2020;67(2):344–53.

12. Zhang Y, Ye Q, He J, Chen P, Wan J, Li J, et al. Recurrence-Associated Multi-RNA Signature to Predict Disease-Free Survival for Ovarian Cancer Patients. Biomed Res Int. 2020;2020:1618527.

13. Chen Y, Zhao XH, Zhang DD, Zhao Y. MiR-513a-3p inhibits EMT mediated by HOXB7 and promotes sensitivity to cisplatin in ovarian cancer cells. Eur Rev Med Pharmacol Sci. 2020;24(20):10391–402.

14. Liu J, Jiang Y, Wan Y, Zhou S, Thapa S, Cheng W. MicroRNA-665 suppresses the growth and migration of ovarian cancer cells by targeting HOXA10. Mol Med Rep. 2018;18(3):2661–8.

15. Zhou P, Xiong T, Yao L, Yuan J. MicroRNA‑665 promotes the proliferation of ovarian cancer cells by targeting SRCIN1. Exp Ther Med. 2020;19(2):1112–20.

16. Vert A, Castro J, Ribó M, Benito A, Vilanova M. Activating transcription factor 3 is crucial for antitumor activity and to strengthen the antiviral properties of Onconase. Oncotarget. 2017;8(7):11692–707.

17. Cai P, Lu Z, Wu J, Qin X, Wang Z, Zhang Z, et al. BTN3A2 serves as a prognostic marker and favors immune infiltration in triple-negative breast cancer. J Cell Biochem. 2020;121(3):2643–54.

18. You Y, Li Y, Li M, Lei M, Wu M, Qu Y, et al. Ovarian cancer stem cells promote tumour immune privilege and invasion via CCL5 and regulatory T cells. Clin Exp Immunol. 2018;191(1):60–73.

19. Pasquier J, Gosset M, Geyl C, Hoarau-Véchot J, Chevrot A, Pocard M, et al. CCL2/CCL5 secreted by the stroma induce IL-6/PYK2 dependent chemoresistance in ovarian cancer. Mol Cancer. 2018;17(1):47.

20. Wu J, Zhang L, Li H, Wu S, Liu Z. Nrf2 induced cisplatin resistance in ovarian cancer by promoting CD99 expression. Biochem Biophys Res Commun. 2019;518(4):698–705.

21. Liu Y, Ding Y, Nie Y, Yang M. EMP1 promotes the proliferation and invasion of ovarian cancer cells through activating the MAPK pathway. Onco Targets Ther. 2020;13:2047–55.

22. Wang Y, Qiu C, Lu N, Liu Z, Jin C, Sun C, et al. FOXD1 is targeted by miR-30a-5p and miR-200a-5p and suppresses the proliferation of human ovarian carcinoma cells by promoting p21 expression in a p53-independent manner. Int J Oncol. 2018;52(6):2130–42.

23. Wang R, Du X, Zhi Y. Screening of Critical Genes Involved in Metastasis and Prognosis of High-Grade Serous Ovarian Cancer by Gene Expression Profile Data. J Comput Biol. 2020;27(7):1104–14.

24. Pinciroli P, Alberti C, Sensi M, Canevari S, Tomassetti A. An IL6-correlated signature in serous epithelial ovarian cancer associates with growth factor response. BMC Genomics. 2013;14(1).

25. Liu X, Gao Y, Zhao B, Li X, Lu YI, Zhang J, et al. Discovery of microarray-identified genes associated with ovarian cancer progression. Int J Oncol. 2015;46(6):2467–78.

26. Tang X han, Li H, Zheng X shuang, Lu M song, An Y, Zhang XL. CRM197 reverses paclitaxel resistance by inhibiting the NAC-1/Gadd45 pathway in paclitaxel-resistant ovarian cancer cells. Cancer Med. 2019;8(14):6426–36.

27. Shen Y, Ruan L, Lian C, Li R, Tu Z, Liu H. Discovery of HB-EGF binding peptides and their functional characterization in ovarian cancer cell lines. Cell Death Discov. 2019;5(1):82.

28. De Andrade WP, Da Conceição Braga L, Gonçales NG, Silva LM, Da Silva Filho AL. HSPA1A, HSPA1L and TRAP1 heat shock genes may be associated with prognosis in ovarian epithelial cancer. Oncol Lett. 2020;19(1):359–67.

29. Nowak M, Glowacka E, Kielbik M, Kulig A, Sulowska Z, Klink M. Secretion of cytokines and heat shock protein (HspA1A) by ovarian cancer cells depending on the tumor type and stage of disease. Cytokine. 2017;89:136–42.

30. Azim SA, Duggan-Peer M, Sprung S, Reimer D, Fiegl H, Soleiman A, et al. Clinical impact of L1CAM expression measured on the transcriptome level in ovarian cancer. Oncotarget. 2016;7(24):37205–14.

31. Bondong S, Kiefel H, Hielscher T, Zeimet AG, Zeillinger R, Pils D, et al. Prognostic significance of L1CAM in ovarian cancer and its role in constitutive NF-κB activation. Ann Oncol. 2012;23(7):1795–802.

32. Shackelford RE, Mayhall K, Maxwell NM, Kandil E, Coppola D. Nicotinamide Phosphoribosyltransferase in Malignancy: A Review. Genes and Cancer. 2013;4(11–12):447–56.

33. Kong B, Wang Q, Fung E, Xue K, Tsang BK. P53 is required for cisplatin-induced processing of the mitochondrial fusion protein L-Opa1 that is mediated by the mitochondrial metallopeptidase Oma1 in gynecologic cancers. J Biol Chem. 2014;289(39):27134–45.

34. Risch HA, Bale AE, Beck PA, Zheng W. PGR +331 A/G and increased risk of epithelial ovarian cancer. Cancer Epidemiol Biomarkers Prev. 2006;15(9):1738–41.

35. Wang M, Wang J, Liu J, Zhu L, Ma H, Zou J, et al. Systematic prediction of key genes for ovarian cancer by co-expression network analysis. J Cell Mol Med. 2020;24(11):6298–307.

36. Kujawa KA, Zembala-Nożyńska E, Cortez AJ, Kujawa T, Kupryjańczyk J, Lisowska KM. Fibronectin and Periostin as Prognostic Markers in Ovarian Cancer. Cells. 2020;9(1):149.

37. Pisanic TR, Cope LM, Lin SF, Yen TT, Athamanolap P, Asaka R, et al. Methylomic analysis of ovarian cancers identifies tumor-specific alterations readily detectable in early precursor lesions. Clin Cancer Res. 2018;24(24):6536–47.
